# Attractive Noncovalent Interactions versus Steric Confinement in Asymmetric Supramolecular Catalysis

**DOI:** 10.1021/jacs.5c17872

**Published:** 2025-12-27

**Authors:** Cristina V. Craescu, Colton D. David, Elizabeth D. Heafner, Kenneth N. Raymond, Robert G. Bergman, F. Dean Toste

**Affiliations:** † Department of Chemistry, 1438 University of California, Berkeley, California 94720, United States; ‡ Chemical Sciences Division, Lawrence Berkeley National Laboratory, Berkeley, California 94720, United States

## Abstract

The remarkable catalytic performance of enzymes stems from their ability to engage in precise noncovalent interactions (NCIs) within a sterically confined space. Supramolecular catalysis seeks to emulate and understand these strategies through the rational design of simple and controlled catalyst microenvironments. While both steric confinement and attractive interactions have been invoked as key to host activity, their relative contribution to rate enhancement and selectivity, as well as potential trade-offs, remains an outstanding question. Here, we address this question by systematically comparing two metal–organic supramolecular catalysts, which differ in the strength of their attractive noncovalent interactions and in their cavity volume. Our findings reveal that the catalyst with the larger cavity, and with stronger available NCIs, exhibits both significant rate acceleration (100-fold) and enhanced enantioselectivity (84% vs 14% ee) in a model ketone reduction compared to its smaller analogue. Mechanistic analysis, binding competition experiments, and computational modeling indicate that these differences predominantly stem from stabilizing noncovalent interactions in the larger catalyst, a result that challenges existing steric-based models of supramolecular stereoinduction. Understanding the governing factors of asymmetric induction and rate acceleration in supramolecular hosts will undoubtedly inform future catalyst design.

## Graphical abstract

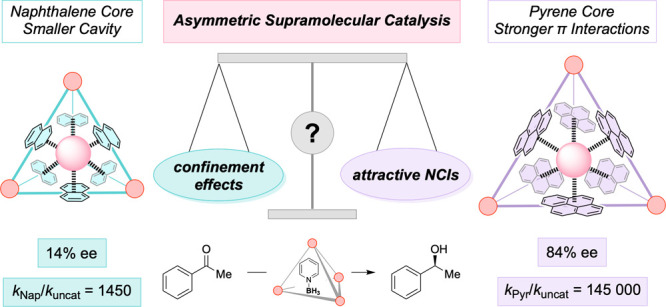

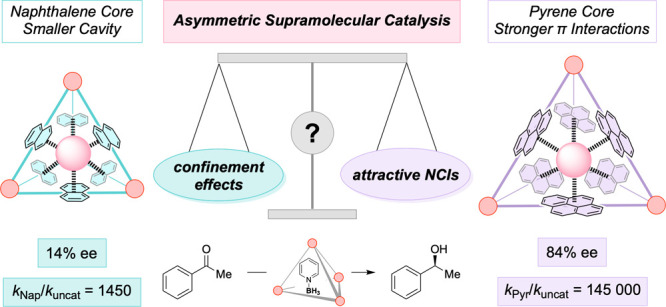

## Introduction

Enzymes leverage noncovalent interactions (NCIs) within precisely tailored active sites to achieve remarkable rate enhancements and enantioselectivities. Their unparalleled catalytic efficiency is often attributed to attractive NCIs—such as electrostatic interactions, hydrogen bonding, π–π, cation−π, and dispersive forces—that are optimally oriented and can act cooperatively to stabilize the favored transition state of a reaction and lower its barrier.^1–5^ In contrast, the design of small-molecule catalysts has largely focused on a fundamentally different approach to achieving selectivity. Rather than stabilizing the desired transition state, small-molecule catalysts are often reported to induce stereoselectivity through repulsive steric interactions with the undesired minor transition state (Figure 1A).^6–9^ More recently, the importance of attractive NCIs in asymmetric catalysis has gained recognition, leading to the development of catalysts that engage substrates through multiple cooperative NCIs in an enzyme-like fashion (Figure 1B,C).^10–25^ Strategies for investigating the mechanisms of acceleration and enantioinduction are well established for both types of these systems and have provided essential guidelines toward catalyst function and design.^12,13,26^

**Figure 1:**
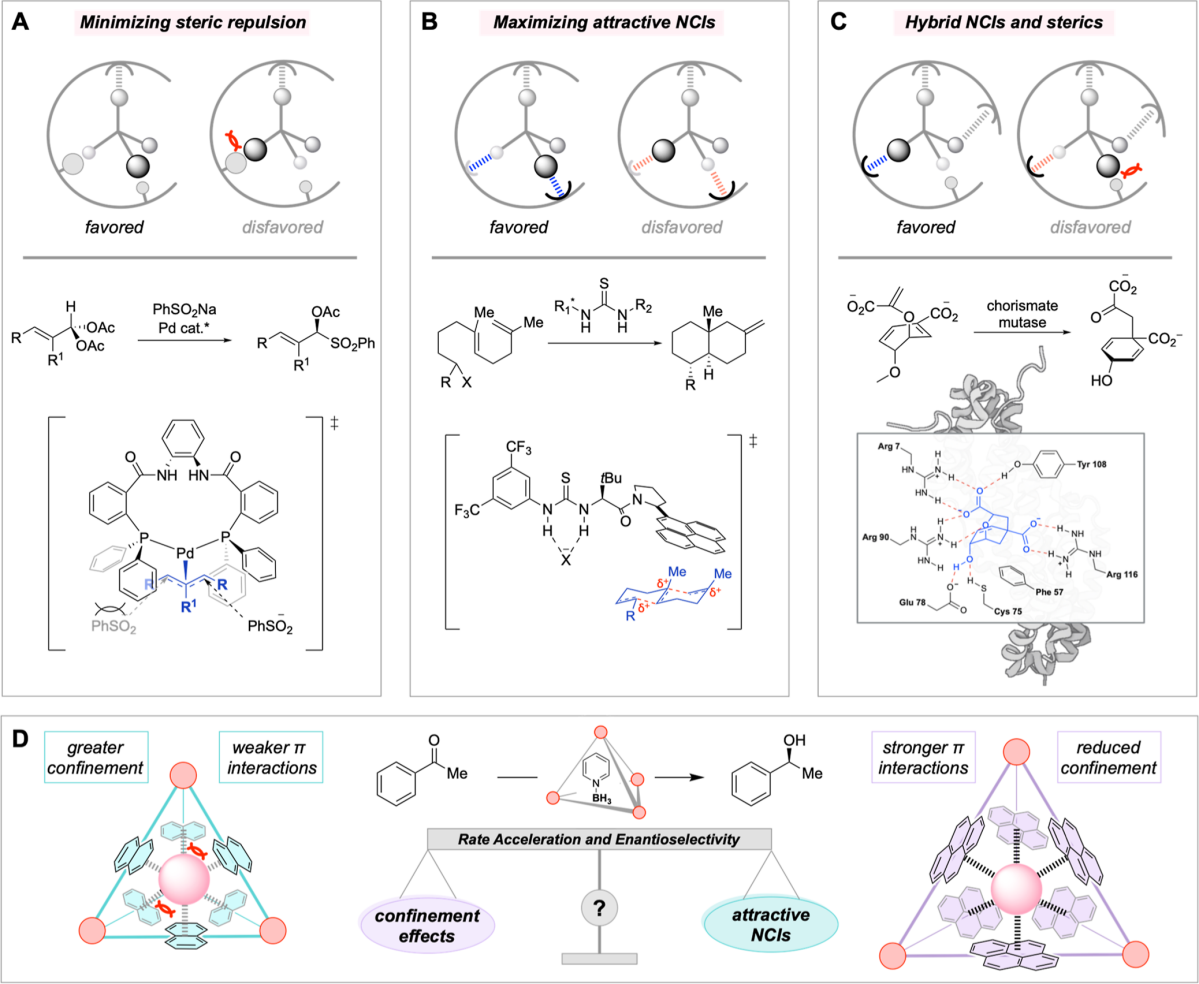
Modes of enantioinduction for small-molecule catalysts and enzymes. (A) Minimizing steric repulsion. (B) Maximizing attractive NCIs. (C) Hybrid model of A and B. This figure was created with BioRender.com. (D) The goals of this work: investigating the trade-off between confinement effects and attractive NCIs for asymmetric supramolecular host catalysis.

Inspired by the catalytic phenomena found in biological systems, supramolecular catalysis seeks to mimic enzyme active sites by introducing a network of NCIs within a confined microenvironment while maintaining the structural simplicity and synthetic accessibility found in small-molecule catalysts.^27–29^ These systems can enable unique reaction pathways and product selectivity distinct from those observed in bulk solution, achieving rate accelerations of up to a billion-fold and enantioselectivities as high as 99% enantiomeric excess (ee).^30–45^ Emerging studies support the importance of attractive NCIs in host recognition and catalysis, but these reports are primarily computational.^46–48^ Despite these advances, in-depth mechanistic studies remain limited and have yet to fully address the trade-off between steric confinement and attractive noncovalent interactions. Such analyses illuminate how both synthetic and enzymatic catalytic processes work and are necessary to establish rational design principles for future supramolecular host catalysts.

The “constrictive binding” model is widely invoked to explain supramolecular enantioinduction. In the model, steric repulsion is held to be responsible for the discrimination between enantiomers; a corollary of the model is that larger host cavities should be less selective due to their reduction in steric confinement.^29,36,49–51^ Here, we report an asymmetric ketone reduction in which a larger supramolecular catalyst facilitates higher levels of enantioselectivity by engaging in attractive noncovalent interactions with guest substrates, rather than relying on constrictive binding. Uncovering how noncovalent interactions operate in supramolecular catalysis can be challenging, owing to their synergistic nature and the limited structural modularity compatible with host self-assembly. To address this, we employ well-established mechanistic tools, previously developed in small-molecule catalysis, to study the relative contributions of steric repulsion and attractive NCIs to rate acceleration and enantioselectivity. Our mechanistic investigation into the mode of enantioinduction in this reaction relies on two supramolecular hosts which differ with respect to both the strength of their attractive noncovalent interactions and their cavity volume (Figure 1D). Eyring analyses on both rate acceleration and selectivity, in concurrence with binding competition studies, detailed kinetic analysis, and computational modeling, reveal the importance of both attractive and repulsive interactions in asymmetric supramolecular catalysis.

## Results and Discussion

### Catalyst Design

The enantiopure Ga_4_L_6_^12–^ Raymond tetrahedron **1** comprises four homochiral (ΛΛΛΛ or ΔΔΔΔ), pseudo-octahedral gallium­(III) vertices, linked by six chiral biscatecholamide ligands (Figure 2A).^52–54^ Bearing an overall 12– charge, the assembly has a unique microenvironment within its confined cavity, exhibiting a high affinity for the binding of cationic guests and enabling stabilization of reactive cationic intermediates and transition states in catalytic processes.^55–57^ In addition, the racemic analogue of this host has demonstrated several features reminiscent of enzymes, such as rate enhancements up to a million-fold and p*K*_a_ shifts of encapsulated guests by as much as 4–5 log units.^31,58^

**Figure 2:**
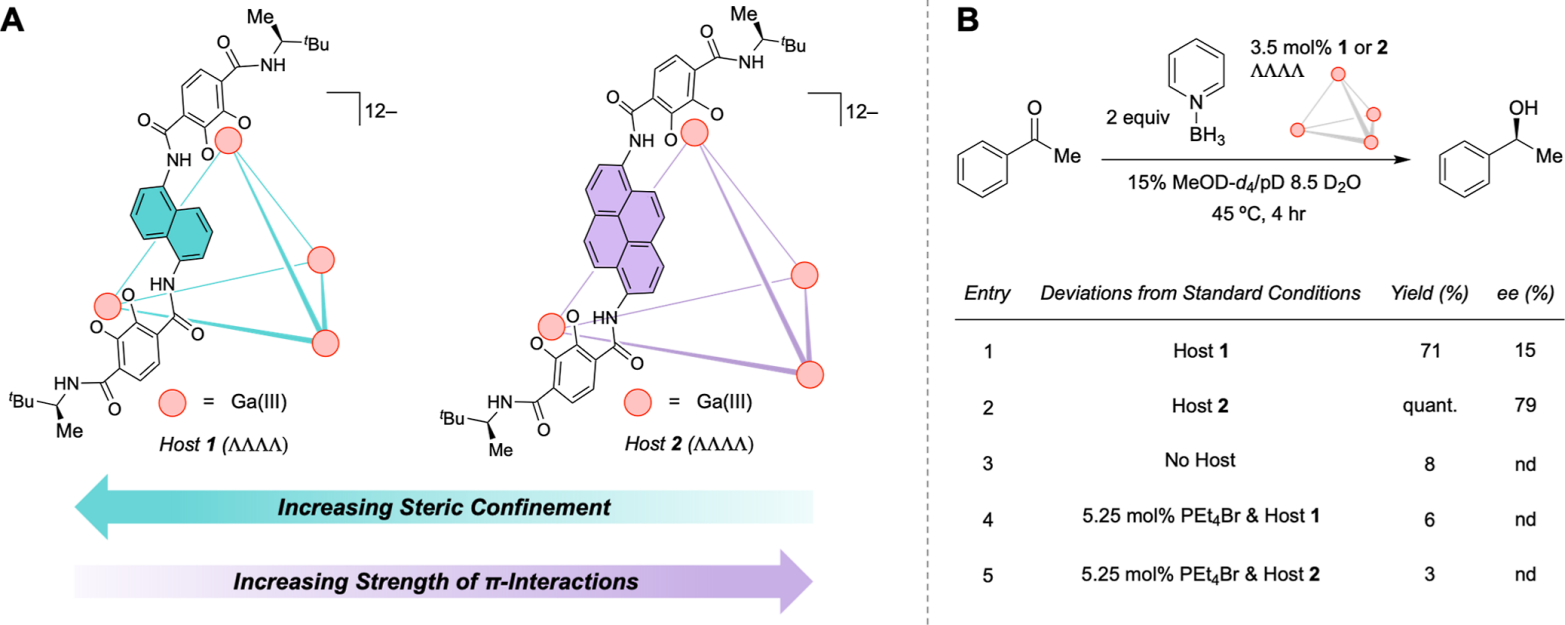
(A) Ga_4_L_6_^12–^ supramolecular hosts **1** and **2**. (B) Control experiments and reaction screening.

Another distinctive feature of host **1** is its chirality. Unlike catalysts that exhibit traditional point or axial chirality, host **1** possesses a helically chiral environment within its cavity, dictated by the chirality of the octahedral metal complexes at its vertices.^54^ While the distal chiral amide groups control the handedness of the vertex complexes, they do not interact directly with the cavity interior. The enantiopure tetrahedral host **1** has been employed in a range of asymmetric organic transformations, demonstrating both high enantioselectivity and rate acceleration.^39,40,50,54^ Host **1** differs from other examples of host-mediated asymmetric catalysis in that the cavity itself serves as both the catalytic site and the source of stereocontrol. In contrast, other systems often rely on either a chiral catalyst encapsulated within an achiral host, or an achiral catalyst operating within a chiral host environment for stereochemical induction.^43,59–61^

Substrate–catalyst interactions play a key role in the stereochemical outcome in asymmetric reactions. Notably, host **1** lacks a conventional “point of contact” and instead encapsulates the entire substrate within its cavity, differentiating it from small-molecule catalysts, where the substrate is typically anchored through multiple well-defined interactions. This absence of inward-facing functional groups might intuitively suggest that stereoinduction and rate acceleration arise primarily from the minimization of steric clashes with the host walls. However, it is important to note that the six naphthalene walls can engage in π interactions such as cation−π and π–π stacking with encapsulated guests. Additionally, a maximum of nine ice-like (i.e., low entropy) water molecules have been shown to occupy the hydrophobic cavity.^62^ This conformationally restricted solvent could therefore act as a directing group and participate in hydrogen bonding with encapsulated substrates, as predicted by recent computational studies.^48,63^

While substrate scope studies have revealed that both steric and electronic factors appear to contribute to host-mediated reactivity, a shift in focus toward catalyst modification is necessary to uncover the general features that govern host-induced stereoselectivity.^40^ To date, modifications to the distal amide groups and changes in the metal identity of **1** have been shown to influence the host’s relative guest exchange rates, which in turn correlate with the enantioselectivity of a model asymmetric reaction.^39^ Core modifications have also been explored, where substituting all the naphthalene spacers on the host walls for pyrenes affords the larger cavity catalyst **2** (Figure 2A).^50,64^ Previous experimental comparison between **1** and **2** led to the proposal that confinement is a dominant factor toward the observed differences in a host-catalyzed Prins reaction.^50^ However, subsequent computational studies contradicted these findings, suggesting that steric constriction cannot solely account for differences in reactivity.^47^

A closer examination of the active site and the NCIs available for **1** and **2** suggests that the aromatic walls lining the cavity may play a more significant role in stereoinduction than previously appreciated. We hypothesized that, due to a combination of increased polarizability, quadrupole moment, and surface area of pyrene in comparison to naphthalene, host **2** might feature stronger cation−π and π–π stacking interactions than **1**.^65–67^ The 1.4-fold increase in cavity volume exhibited in host **2** relative to host **1** might also accommodate more conformationally restricted solvent molecules capable of hydrogen bonding.^52,64^ A comparison of hosts **1** and **2** thus serves as a means of investigating the contributions of steric confinement and attractive NCIs to rate enhancement and selectivity in supramolecular catalysis.

### Reaction Development

Host-mediated reductions of imines, aldehydes, and oximes using a pyridine borane cofactor, via acid catalysis, are well precedented with enantiopure host **1** and its racemic variant.^40,68^ Previous work on the host-mediated reduction of oximes to hydroxylamines demonstrated the potency of **1** as an enantioselective catalyst, affording products with up to 99% ee.^40^ Interestingly, the asymmetric reduction of analogous ketones with **1** resulted in poor enantioselectivity despite the structural similarity between substrates. While acetophenone (**a**) furnished the corresponding alcohol product in 71% yield and 15% ee in the presence of host **1** (smaller cavity, weaker NCIs), host **2** (larger cavity, stronger NCIs) resulted in quantitative product formation and a much improved 79% ee (Figure 2B). Control experiments showed that background reactivity was suppressed at a D_2_O basicity level (pD) of 8.5. Upon addition of PEt_4_Br, a strongly bound competitive inhibitor, minimal product was observed, confirming that the catalysis occurs within the cavity interior rather than its exterior (Figure 2B).^29^ Thus, the differences in product yield and enantioselectivity of alcohol **a** afforded by hosts **1** and **2** can be meaningfully interpreted as a consequence of their distinct microenvironments. This preliminary result of a large selectivity difference between hosts **1** and **2** motivated a deeper mechanistic study of host stereoinduction.

To better understand the generality of this reactivity and selectivity difference, the reaction was examined with selected substrates by using both host catalysts (Figure 3). Among structural modifications, substrate fluorination was employed to study electron-deficient substrates with minimal added steric bulk. Increasing the fluorination of substrates **a**–**c** resulted in overall lower yields. Decreased reactivity is consistent with a more unfavorable protonation for electron-deficient substrates.

**Figure 3:**
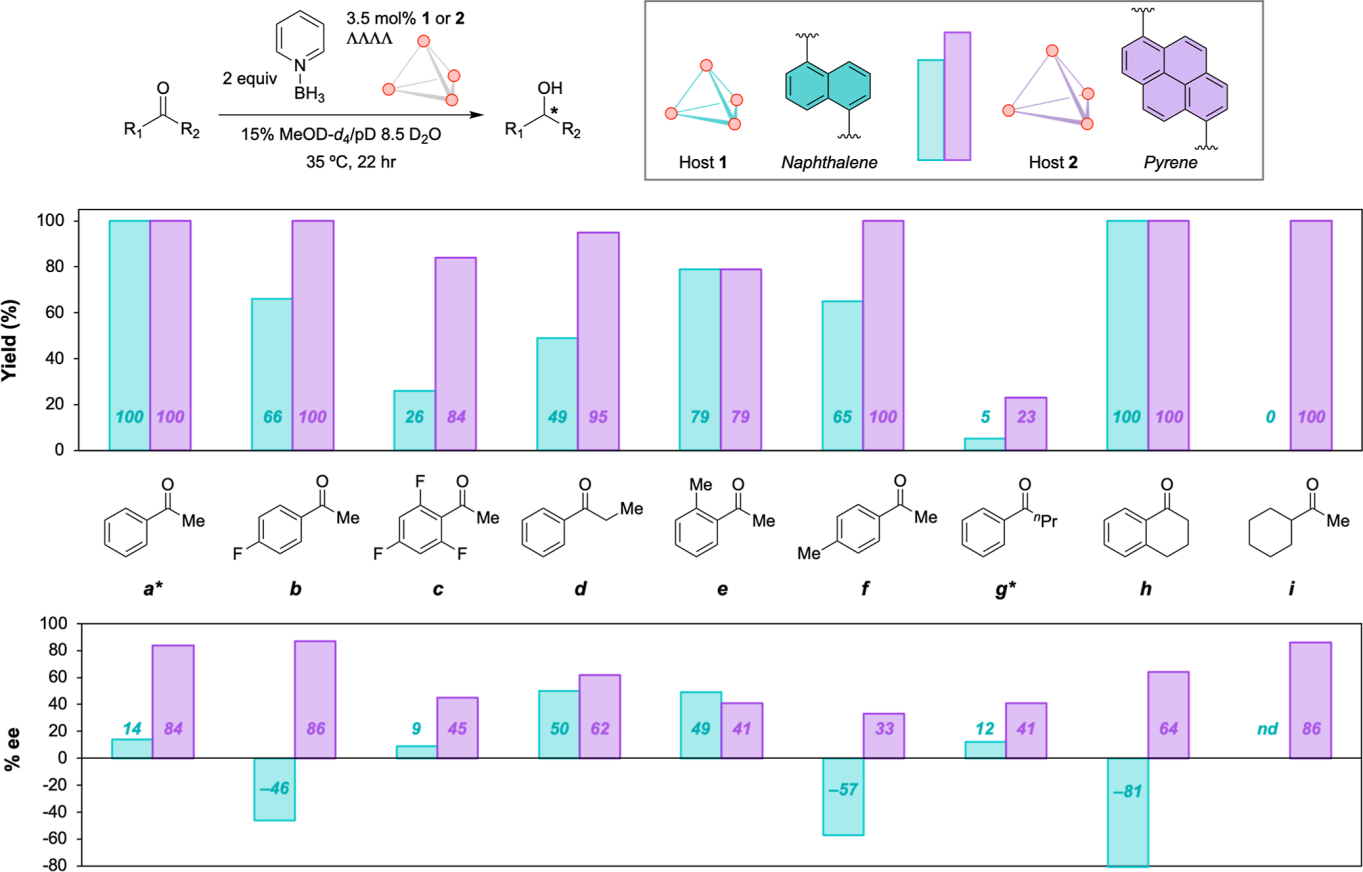
Yield and % ee achieved in the reduction of ketones **a**–**i** with both host catalysts (*reaction stopped after 24 h).

Compared to **a**, the regioisomeric substrates **d**–**f**, featuring a methyl group at varying positions on the aryl ring, displayed diminished selectivity differences between **1** and **2**. While the enantioselectivity of the smaller host **1** appears to benefit from these more sterically encumbered substrates, the opposite trend is observed for host **2**, suggesting that steric repulsion is not a dominant factor in the enantioselectivity of host **2**.

Subjecting substrate **h**, which can be viewed as a cyclic congener of substrate **g** with the alkyl chain tied back, to the reaction condition resulted in increased yields and selectivity with both catalysts, which we propose is due to more favorable interactions with the cavity walls. Lastly, aliphatic ketone **i** showed no detectable reactivity with **1**, while **2** afforded the desired alcohol with full conversion and 86% ee. The comparable yields and selectivity of substrates **a** and **i** with host **2** support a common underlying interaction, distinct from that of host **1**.

Across the series, host **2** (stronger NCIs) resulted in improved yields and enantioselectivity relative to host **1** (smaller cavity), with minor exceptions. These findings contradict the classical notions that lower volume, and therefore more sterically confined spaces, are crucial for enantioinduction.

### Kinetic Analysis and Binding Competitions

An understanding of the host-catalyzed mechanism is critical for the assessment of underlying interactions. The analogous reduction of oximes with pyridine borane in the presence of catalyst **1** has been previously studied.^40^ Kinetic analysis revealed a first-order dependence on both the host and the substrate, with saturation kinetics observed in the reductant cofactor above 1 equiv.^40^

To assess how these findings translate to the ketone reductions, a series of ^19^F NMR kinetics experiments with substrate **b** and hosts **1** and **2** were performed. The reaction was found to exhibit a pseudo-first-order dependence on substrate with saturation in pyridine borane. A primary kinetic isotope effect (KIE) was observed for both **1** and **2** in parallel reactions using pyridine proteoborane (BH_3_) and deuteroborane (BD_3_), suggesting that hydride delivery is the rate-determining step (Figure 4A,B). Overall, we postulate that the host-catalyzed ketone reduction proceeds through a reversible, fast encapsulation of the substrate and subsequent protonation, followed by rate-limiting irreversible delivery of the hydride to furnish the alcohol product (Figure 4B). Enantiodiscrimination at the host aperture is unlikely, as indicated by the similar guest exchange rates observed for model *R* and *S* chiral ammonium salts.^40^ Additionally, minimal thermodynamic differentiation between these model enantiomers suggests that binding is not stereoselective.^40^ Therefore, it can be concluded that the hydride delivery step is rate- and selectivity-determining, proceeding under Curtin–Hammett control from rapidly equilibrating ketone–reductant–host complexes.^69^

**Figure 4:**
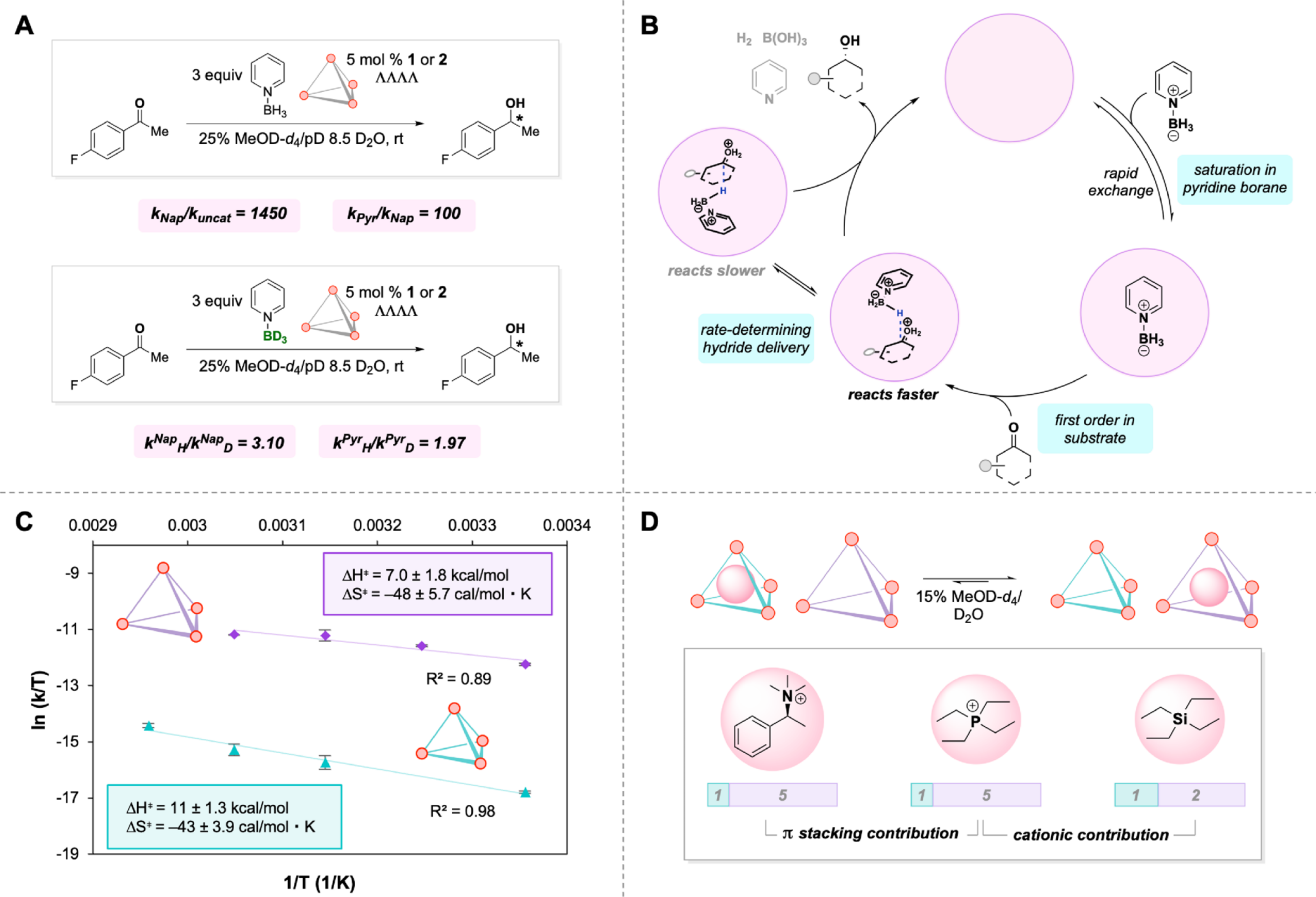
(A) Rate acceleration and KIE experiments. (B) Proposed mechanism for host-catalyzed reductions. (C) Eyring analysis on reaction rates with **1** and **2**. (D) Binding competition experiments.

To meaningfully interpret the observed differences in selectivity and activity between **1** and **2**, the host catalysis must be sufficiently rapid to outcompete the uncatalyzed background (racemic) reactivity. Under optimized conditions, 1450- and 145,000-fold rate accelerations were observed for hosts **1** and **2**, respectively, relative to the background reaction: a striking 100-fold rate enhancement for host **2** relative to its more confined analogue **1**.

To determine whether this pronounced rate discrepancy is enthalpic or entropic in origin, the temperature dependence of the reaction rates was examined. Notably, the extracted pseudo-first-order rate constant representing overall catalytic efficiency is dependent on hydride delivery, as well as preceding substrate binding and protonation (see Supporting Information). Variable temperature kinetic measurements revealed a 4 kcal/mol difference in activation enthalpy (Δ*H* ^⧧^) between hosts **1** (11 ± 1.3 kcal/mol) and **2** (7 ± 1.8 kcal/mol), while the activation entropies (Δ*S* ^⧧^) are the same within error (Figure 4C). If substrate preorganization was a dominant contributor, then a lower Δ*S* ^⧧^ would be expected for the more confined host **1** relative to **2**. While steric repulsion could be contributing to an increased Δ*H* ^⧧^ in the smaller catalyst **1**, the enthalpic difference between the two hosts is unlikely to arise from steric interactions alone, given the similarity of the activation entropies observed. Thus, we hypothesized that the enhanced reactivity and selectivity observed with host **2** could be attributed to stronger NCIs in the cationic transition state, giving rise to the decreased Δ*H* ^⧧^ observed with **2** compared to **1**.

To further probe the specific NCIs responsible for the markedly different reactivities, guest binding competitions were performed (Figure 4D). Host–guest competition experiments consist of equilibrated equimolar mixtures of two different hosts and one guest. The ratio of the resulting host–guest complexes serves as a thermodynamic probe for the guest’s binding preference. When hosts **1** and **2** compete for PEt_4_^+^, a significant preference of 1:5 in favor of host **2** was observed. In contrast, when its neutral isostere SiEt_4_ is tested, the ratio shifted to only 1:2, corresponding to a more modest bias. Notably, the 1:5 binding preference for host **2** was retained, even when a larger benzylammonium salt was used. Overall, these results suggest that the pyrene-based host **2** is a stronger receptor for cations than its naphthalene analogue **1**. This enhanced binding cannot be attributed solely to guest size or solvent exclusion effects, as the neutral SiEt_4_, which is essentially equal in size to PEt_4_^+^, displays a significantly reduced preference for host **2**. Instead, we hypothesized that the increased affinity of cationic guests for host **2** arises from stronger cation−π interactions, consistent with the well-precedented preference of larger arenes to engage cations due to their increased polarizability.^67^ Therefore, catalyst **2**’s pronounced affinity for cations likely contributes to the observed decrease in activation barrier in the rate-determining step.

Overall, the ketone reduction mechanism features a buildup of positive charge in the hydride-delivery transition state and in the preceding intermediates. We propose that the transient cationic species involved in catalysis are stabilized more significantly by host **2** due to stronger cation−π interactions, resulting in the decreased enthalpic barrier and increased reaction rate compared to **1**.

### Eyring Analysis on Selectivity

To further investigate the observed differences in enantioinduction achieved with the two catalysts, an Eyring analysis of enantioselectivity was conducted. Inspired by work from Jacobsen and co-workers,^11,12,26^ the enantiomeric ratio (er) was measured at different temperatures to extract the differential enthalpic (ΔΔ*H* ^⧧^) and entropic (ΔΔ*S* ^⧧^) parameters of diastereomeric transition states. This analysis aims to gain insight into the NCIs responsible for enantioinduction within hosts **1** and **2** by examining relative trends in enthalpic contributions. Standard host-catalyzed reductions were conducted between 25 and 65 °C (see Supporting Information) to obtain the temperature dependence of er for each substrate (**a**–**d**) and catalyst pair (Figure 5A).

**Figure 5:**
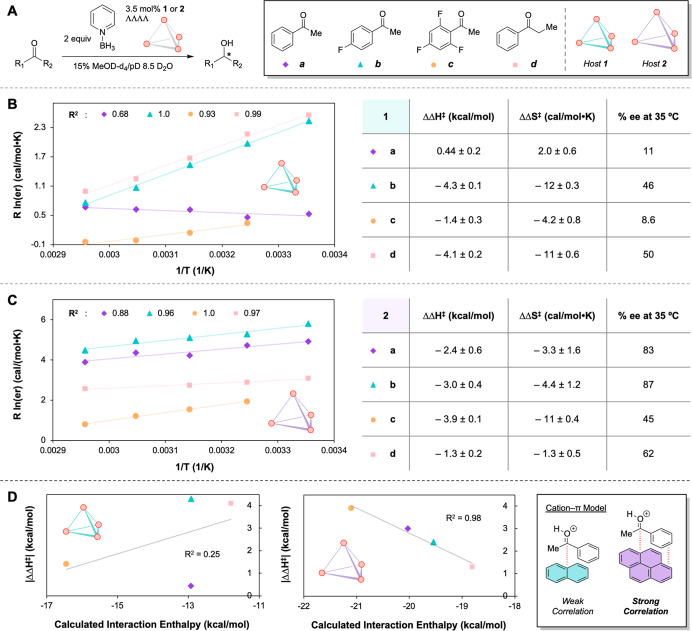
(A) Reaction conditions and selected substrates for Eyring analysis on selectivity. (B) Extracted differential activation parameters for host **1**. (C) Extracted differential activation parameters using host **2**. (D) Correlation between |ΔΔ*H* ^⧧^| and calculated interaction enthalpy.

The reduction of ketone **a** with **1** resulted in a small, unfavorable ΔΔ*H* ^⧧^, as compared to the larger, negative ΔΔ*H* ^⧧^ for host **2** (Figure 5B,C). This difference in the enthalpic contribution to ee between **1** and **2** (2.8 kcal/mol) is difficult to attribute to repulsive steric effects alone, implying that stabilizing NCIs may be significant in the favored transition state.^18^ While selectivity in the reduction of **a** shows different driving forces between the two catalysts, host **2** is better able to enthalpically select for the major diastereomeric pathway compared to **1**, consistent with the presence of strong NCIs.

Ketones **b** and **c** were then chosen to investigate the impact of fluorination on the selectivity. Fluorination renders the substrate more electron-deficient in the selectivity-determining transition state, which should enhance its interaction strength with the host walls, an effect most evident in changes in ΔΔ*H* ^⧧^ across **a**–**c**. While no clear trend in enthalpy was observed with **1** (Figure 5B), the magnitude of ΔΔ*H* ^⧧^ achieved with host **2** (Figure 5C) increased with the extent of fluorination.

To elucidate the interactions contributing to the selectivity, a simplified computational model was developed. Specifically, precedented Density Functional Theory (DFT) methods were used to calculate the interaction energies between protonated substrates and either a naphthalene or pyrene unit representing truncated host walls.^70^ The experimental |ΔΔ*H* ^⧧^| values for substrates **a**–**c** displayed a strong correlation with the calculated interaction enthalpies for host **2**, while no correlation was observed for **1** (Figure 5D). Notably, no correlation was obtained between |ΔΔ*G* ^⧧^| and the interaction free energy, highlighting the insight provided by an Eyring analysis on selectivity (see Supporting Information). While the model accounts for both cation−π and π–π interactions in the gas phase, the former is expected to play a more significant role. This hypothesis is consistent with the ΔΔ*H* ^⧧^ values obtained for aliphatic ketone **i** and **a** being identical within error (see Supporting Information). While these results do not exclude the contribution of other relevant NCIs such as substrate-solvent hydrogen bonding, they highlight the importance of host-substrate cation−π interactions in the effective enantioinduction within host **2**.

Substrate **d** was selected for Eyring analysis to explore the effect of increased steric bulk proximal to the reactive carbonyl moiety, particularly because this substrate afforded one of the smallest selectivity differences between **1** and **2** (Figure 3). The ΔΔ*H* ^⧧^ values obtained for **1** and **2** indicate that the smaller catalyst **1** benefits from a much larger differential enthalpic stabilization than that of the bigger host **2**. It is hypothesized that the added bulk on the substrate allows **1** to better discriminate between the two diastereomeric transition states in a steric-controlled manner. Steric bulk in the rate- and selectivity-determining transition states should increase the magnitude of both Δ*H* ^⧧^ and ΔΔ*H* ^⧧^. This hypothesis is consistent with the reduced yield but higher % ee resulting from ketone **d** compared to **a** with catalyst **1** (Figure 3). Additionally, regioisomers of ketone **d**, **e**, and **f** resulted in better enantioselectivity with **1** than with **2**, supporting the substrate-controlled steric bulk being required for greater chiral discrimination in **1**. In contrast, the ethyl group may interfere with the alignment and proximity of cation−π interactions necessary for efficient enantioinduction in **2**. This proposal is consistent with **d** having the weakest calculated interaction with pyrene of ketones **a**–**d** while following the correlation between the interaction strength and |ΔΔ*H* ^⧧^| for host **2**.

Notably, the variation in selectivity across substrates exhibits compensatory effects with **1** and **2**, such that for an increasingly favorable ΔΔ*H* ^⧧^, an opposing response in ΔΔ*S* ^⧧^ is observed (Figure 5B,C; see Supporting Information). However, **2** strikes an overall improved balance of both parameters and imparts greater enantioinduction than **1** across the majority of substrates.

Overall, the enthalpic contributions to selectivity were extracted for catalysts **1** and **2** in the reductions of substrates **a**–**d**. The strong correlation with interaction energies observed for **2**, concurrent with the lack thereof for **1**, supports different modes of enantioinduction between the two hosts. While the selectivity achieved with the small-cavity host **1** is highly influenced by the steric profile of the substrates, its larger analogue relies more on attractive cation−π interactions in the favored transition state. These findings showcase the potential for supramolecular catalysts to access both NCI- and sterically controlled regimes of enantioinduction, much like enzymes.^71,72^

## Conclusion

The contributions of attractive noncovalent interactions and repulsive steric interactions to supramolecular rate acceleration and enantioselectivity were investigated through the lens of a novel asymmetric ketone reduction. Detailed kinetic analysis revealed a significant reduction in activation enthalpy for host **2** (larger cavity, stronger NCIs), leading to 100-fold rate enhancement compared to host **1** (smaller cavity, weaker NCIs). Binding competitions support a markedly increased affinity for cations in pyrene-based host **2**, compared to that of its naphthalene analogue **1**. The less confined cavity of **2** affords higher selectivity across substrates, with selectivity positively correlating with the strength of the noncovalent interactions between the host walls and the catalytic intermediate. In contrast, the smaller host catalyst **1** operates in a sterically controlled regime, achieving higher enantioselectivity than **2** only with bulkier substrates. While enhanced stabilizing interactions can lead to both greater rate enhancements and selectivity, steric control in a more confined host necessarily requires a trade-off of the two. This report aims to guide the rational design of new asymmetric supramolecular catalysts, bringing to light the interplay of NCIs and steric interactions responsible for catalytic activity and selectivity.

## Supplementary Material



## Notes

General procedures; synthesis and characterization of compounds; representative ^1^H NMR and ^19^F NMR spectra; kinetic profile and analysis; Eyring analysis on selectivity; HPLC traces; and coordinates for DFT-optimized structures (PDF)
