# Supplementary material for: Attractive Noncovalent Interactions versus Steric Confinement in Asymmetric Supramolecular Catalysis
Source: J Am Chem Soc. 2025 Dec 27;148(1):1306–15. doi: 10.1021/jacs.5c17872 (PMC12814166; doi:10.1021/jacs.5c17872)
Supplement: Supplementary file 1 [file ja5c17872_si_001.pdf]

Attractive Noncovalent Interactions versus Steric Confinement in Asymmetric Supramolecular Catalysis

Authors: Cristina V. Craescu,[1,2,§] Colton D. David,[1,2,§] Elizabeth D. Heafner,[1,2] Kenneth N. Raymond,[1,2] Robert G. Bergman,[1,2] and F. Dean Toste[1,2*]

Affiliations and Correspondence:
[1]Department of Chemistry, University of California, Berkeley, California 94720, United States
[2]Chemical Sciences Division, Lawrence Berkeley National Laboratory Berkeley, California 94720, United States
[§]Authors contributed equally
*Correspondence: *fdtoste@berkeley.edu*

Table of Contents

General Methods and Instrumentation **3**
Synthesis and Characterization of Compounds **4**
Ligand Synthesis 4
Ligand (S)-S3 synthesis general scheme *4*
Tetramethyl protected biscatechol-ligand (S)-S2 .... *4*
Ligand (S)-S3 .... *4*
Host Synthesis 5
Binding Competition Experiments **6**
Procedures and Conditions for Ketone Reduction Conditions **8**
General Procedure A for Racemic Ketone Reductions 8
General Procedure B for Host-Catalyzed Ketone Reductions 8
General Procedure C for Racemic Ketone Functionalization 9
General Procedure D for Enantioenriched Ketone Functionalization 9
Representative NMR Spectra 9
Ketone Reduction Kinetics and Mechanistic Investigation **16**
General Procedure for Kinetics Experiments 16
Example Reaction Profiles 17
Extracted Rate Constants 19

Mechanistic Discussion of k_{obs} 20
Eyring Analysis on Enantioselectivity **22**
General procedure for Eyring Analysis Reductions 22
Differential Enthalpy–Entropy Compensation 24
Eyring Analysis on Rates 24
NMR Spectra **26**
Computational Workflow **28**
Interaction Energies and Correlation Data 29
XYZ Coordinates for Interaction Energy Calculations 30
Chiral HPLC Traces **45**
Supplemental References **53**

General Methods and Instrumentation

All reactions were capped and performed at room temperature under ambient atmosphere with Teflon-coated magnetic stir bars unless otherwise specified. Air sensitive materials were prepared using standard Schlenk technique and handled in a wet glovebox under a nitrogen (N_2) atmosphere. All solvents were degassed for 0.5 hours by sparging before introduction into the glovebox. Flash column chromatography was performed on a Teledyne Isco CombiFlash Rf instrument using pre-packed silica gel columns unless otherwise stated. Thin layer chromatography (TLC) analysis was performed using Merck 60 pre-coated silica gel plates with F254 indicator and visualized using UV light (254 nm) or potassium permanganate ($KMnO_4$). Commercial grade solvents were used without further purification except as indicated. Dichloromethane (CH_2Cl_2), diethylether (Et_2O), tetrahydrofuran (THF), and triethylamine (Et_3N) were dried by passing the previously degassed solvents through activated alumina columns under argon. All other reagents were used directly as received without further purification. All deuterated solvents were used as received from Cambridge Isotope Laboratories, Inc. Proton nuclear magnetic resonance (^1H NMR), carbon nuclear magnetic resonance (^{13}C NMR), and fluorine nuclear magnetic resonance (^{19}F NMR) spectra were taken with Bruker AVQ-400, NEO-500, NEO-501, AV-600, or AV-700 and JEOL-400 NMR spectrometers operating at 400 MHz, 500 MHz, 600, or 700 MHz. Chemical shifts are reported in parts per million (ppm) with reference to the appropriate residual solvent signal. ^1H NMR: $CDCl_3$: δ: 7.26 ppm, CD_2Cl_2: δ: 5.32 ppm, DMSO-d_6: δ: 2.50 ppm, MeOD-d_4: δ: 3.31 ppm, D_2O: δ: 4.79 ppm. ^{13}C NMR: $CDCl_3$: δ: 77.16 ppm, CD_2Cl_2: δ: 53.84 ppm, DMSO-d_6: δ: 39.52 ppm, MeOD-d_4: δ: 49.00 ppm. ^1H NMR multiplicities are reported as (s = singlet, d = doublet, t = triplet, q = quartet, quint = quintet, hept = heptet, oct = octet, m = multiplet; coupling constant(s) in Hz; integration). High resolution mass spectra were recorded by an Agilent 6230 ESI-TOF LC/MS and are given in m/z. The determination of enantiomeric excess was performed by chiral phase high performance liquid chromatography (HPLC) using Shimadzu Prominence HPLC and Diacel ChiralCel OJ-H, AD-H, IB, IC, and IG columns. Samples for chiral HPLC analysis were prepared in 95% hexanes/isopropanol (IPA) solutions unless otherwise stated. M_4L_6 hosts were stored in a wet glovebox to avoid oxidative decomposition.

Synthesis and Characterization of Compounds

Ligand Synthesis

Ligand (*S*)-S3 synthesis general scheme

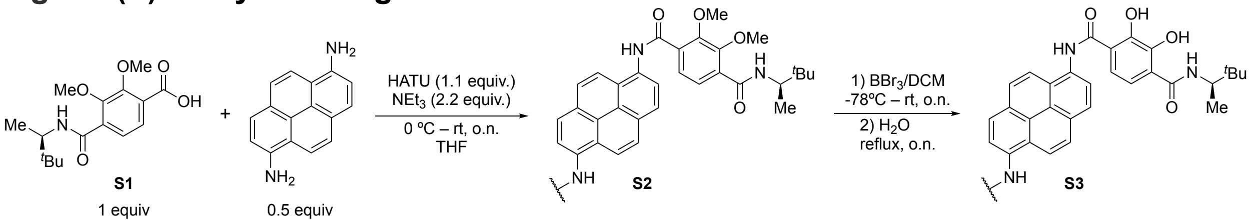

Tetramethyl protected biscatechol-ligand (*S*)-S2

Chiral acid **S1** (1.3 g, 4.3 mmol, 1.0 equiv.), Et_3N (5.6 mL, 9.5 mmol, 2.2 equiv.) and HATU (1.8 g, 4.7 mmol, 1.1 equiv.) were suspended in THF (50 mL) at 0 °C and stirred for 1 hour. Diaminopyrene (0.5 g, 2.1 mmol, 0.5 equiv.) was then added portion wise to the ice-cold solution. The resulting mixture was allowed to warm slowly to room temperature and stirred for 20 hours. The crude reaction mixture was concentrated in vacuo, redissolved a minimum amount of THF (30 mL), sonicated and cooled to 0 °C. The resulting pale-yellow solid was filtered, washed with 1M HCl and Et_2O to afford title compound in 71% yield (1.2 g).

^{1}H NMR (500 MHz, $CDCl_3$) δ 10.66 (s, 1H), 8.87 (d, *J* = 8.4 Hz, 1H), 8.16 (d, *J* = 8.4 Hz, 1H), 8.14 – 8.03 (m, 3H), 7.98 (d, *J* = 8.4 Hz, 1H), 7.77 (d, *J* = 9.4 Hz, 1H), 4.19 (s, 3H), 4.14 – 4.08 (m, 1H), 4.05 (s, 3H), 1.16 (d, *J* = 6.8 Hz, 3H), 0.97 (s, 9H).

^{13}C NMR (126 MHz, $CDCl_3$) δ 163.43, 162.70, 151.67, 151.51, 131.72, 131.37, 129.75, 128.47, 128.37, 127.23, 127.20, 125.67, 125.63, 122.69, 121.44, 118.87, 62.56, 62.15, 53.55, 34.45, 26.53, 16.28.

MS (ESI-MS, pos) (m/z): [$C_{48}H_{54}N_4O_8+H^+$] = Theoretical: 815.4014; Observed: 815.3986.

Ligand (*S*)-S3

Protected ligand **S3** (1.2 g, 1.6 mmol) was dissolved in DCM (100 mL) and cooled to −78 °C, followed by addition of BBr_3 (2 mL). The resulting red suspension was allowed to warm slowly to room temperature and stirred overnight. The reaction mixture was then poured onto 100 g of ice and allowed to warm to room temperature. The resulting slurry was filtered to give a yellow solid, which was then suspended in water (100 mL) and heated at reflux for 16 h. The suspension was cooled to room temperature, filtered, dried on the frit, and used without further purification. Deprotected ligand **S3** was isolated as a white powder in 65 % yield (0.74 g).

^{1}H NMR (500 MHz, DMSO-d_6) δ 8.50 (d, J = 9.3 Hz, 1H), 8.45 (d, J = 8.2 Hz, 1H), 8.40 (d, J = 8.4 Hz, 1H), 8.28 (q, J = 9.3 Hz, 2H), 7.72 (d, J = 8.7 Hz, 1H), 7.65 (d, J = 8.7 Hz, 1H), 4.07 (dq, J = 9.1, 6.9 Hz, 1H), 1.16 (d, J = 6.9 Hz, 3H), 0.95 (s, 9H).

^{13}C NMR (126 MHz, DMSO-d_6) δ 167.76, 167.09, 149.74, 148.65, 131.21, 128.76, 127.75, 125.35, 124.72, 124.47, 124.18, 121.47, 119.32, 118.26, 117.20, 52.56, 34.68, 30.72, 26.35, 15.37.

MS (ESI-MS, pos) (m/z): [$C_{44}H_{46}N_4O_8+H^+$] = Theoretical: 757.3243; Observed: 757.3236.

Host Synthesis

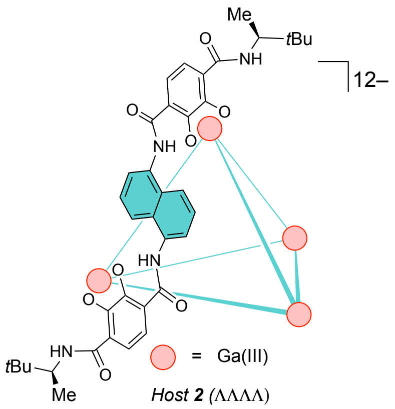

$K^{12}[Ga_4L^*_6]$ Host 1 was synthesized according to a reported procedure.[1]

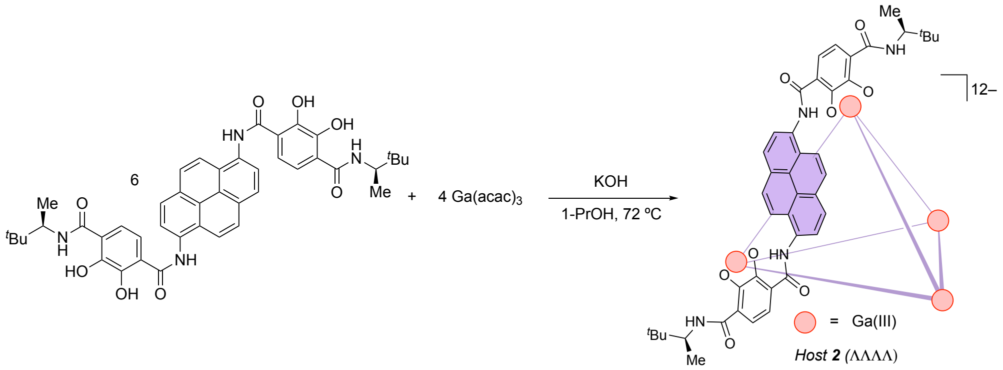

$K^{12}[Ga_4L^*_6]$ Host 2: Ligand **S3** (61.2 mg, 0.081 mmol, 6 equiv.) and $Ga(acac)_3$ (20 mg, 0.055 mmol, 4 equiv.) were charged to a 25 mL round bottom flask equipped with a stir bar. The flask was evacuated and backfilled 3x with N_2, after which degassed 95:5 1-propanol/H_2O (4 mL) was added, and the mixture was sparged for another 15 min. The yellow suspension was heated to 72°C and allowed to stir for 30 minutes, after which a color change to orange is observed. Then, KOH (0.15 mmol, 11.4 equiv., 0.11 M) was added as a degassed solution in 95:5 1-propanol/H_2O (1.4 mL) at 0.025 mL/min via a syringe pump. After base addition, the homogenous mixture was allowed to stir at 72°C

stir for 2 more hours. The reaction was removed from the heating source and diethyl ether (15 mL) was immediately added to the hot solution. The resulting precipitate was then filtered over a fine glass frit and washed 2x with diethyl ether (10 mL). The supramolecular assembly product **2** was collected as a yellow solid (63 mg, 0.012 mmol, 88% yield) and stored in an N_2 wet box.

Binding Competition Experiments

PEt_4Br Competition: Stock solutions of supramolecular host **1** (10 mM), **2** (5 mM), and PEt_4Br (60 mM) in D_2O were added to an NMR tube and diluted to a final concentration of 1 mM each in 15% MeOD-d_4:D_2O. The resulting mixture was allowed to equilibrate for 2 hours prior to measurement. (d1=15s)

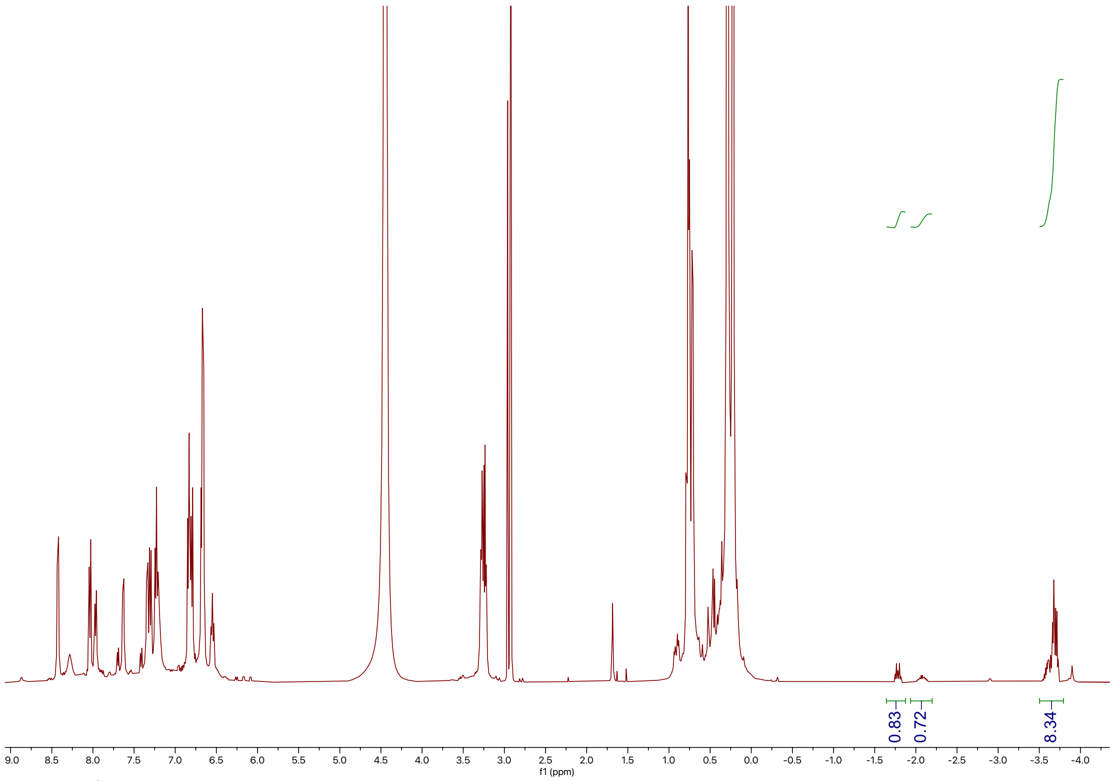

Figure S1. ^1H NMR spectrum of the equimolar PEt_4Br:**1**:**2** competition mixture. Encapsulated guest peaks are integrated for clarity, with the more upfield signals being guest bound to host **2**.

(*R*)-$BnNMe_3I$ Competition: Stock solutions of supramolecular host **1** (10 mM), **2** (5 mM), and (R)-$BnNMe_3I$ (63 mM) in D_2O were added to an NMR tube and diluted to a final concentration of 1 mM each in 15% MeOD-d_4:D_2O. The resulting mixture was allowed to equilibrate for 2 hours prior to measurement. (d1=15s)

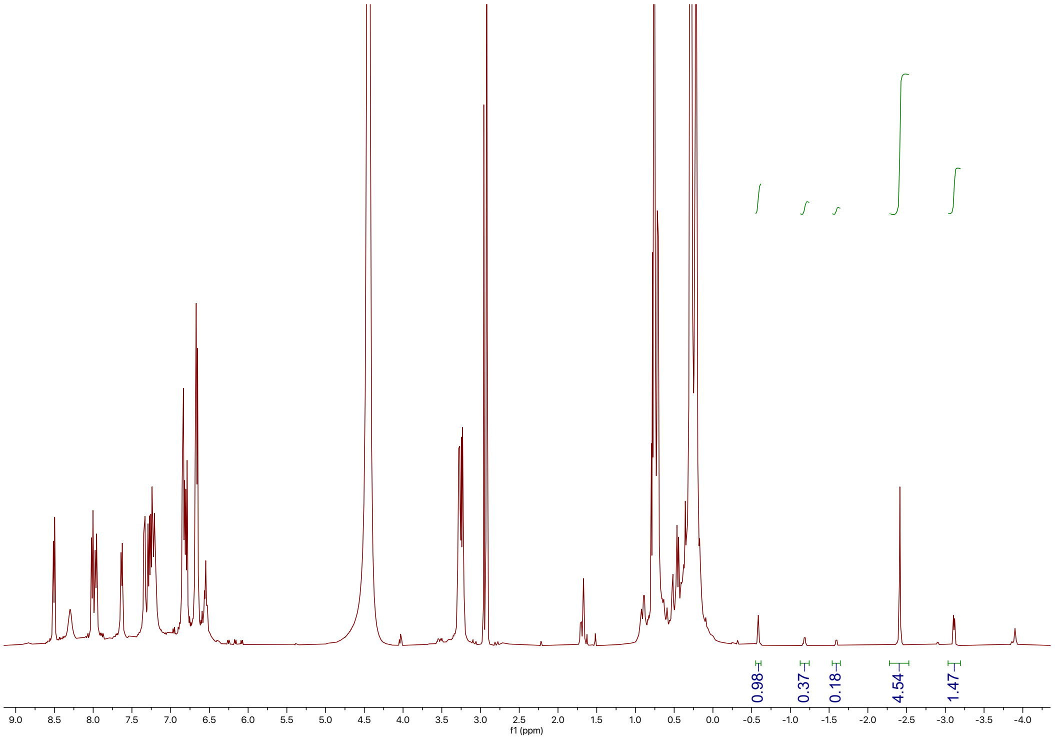

Figure S2. ^{1}H NMR spectrum of the equimolar (*R*)-$BnNMe_3I$:**1**:**2** competition mixture. Encapsulated guest peaks are integrated for clarity, with the more upfield signals being guest bound to host **2**.

$SiEt_4$ Competition Stock solutions of supramolecular host **1** (4.9 mM), **2** (12 mM), and $SiEt_4$ (28.5 mM) in MeOD-*d*$_4$ were added to an NMR tube and diluted to a final concentration of 1 mM each in 15% MeOD-*d*$_4$:D_2O. The resulting mixture was allowed to equilibrate for 2 hours prior to measurement. (d1=15s).

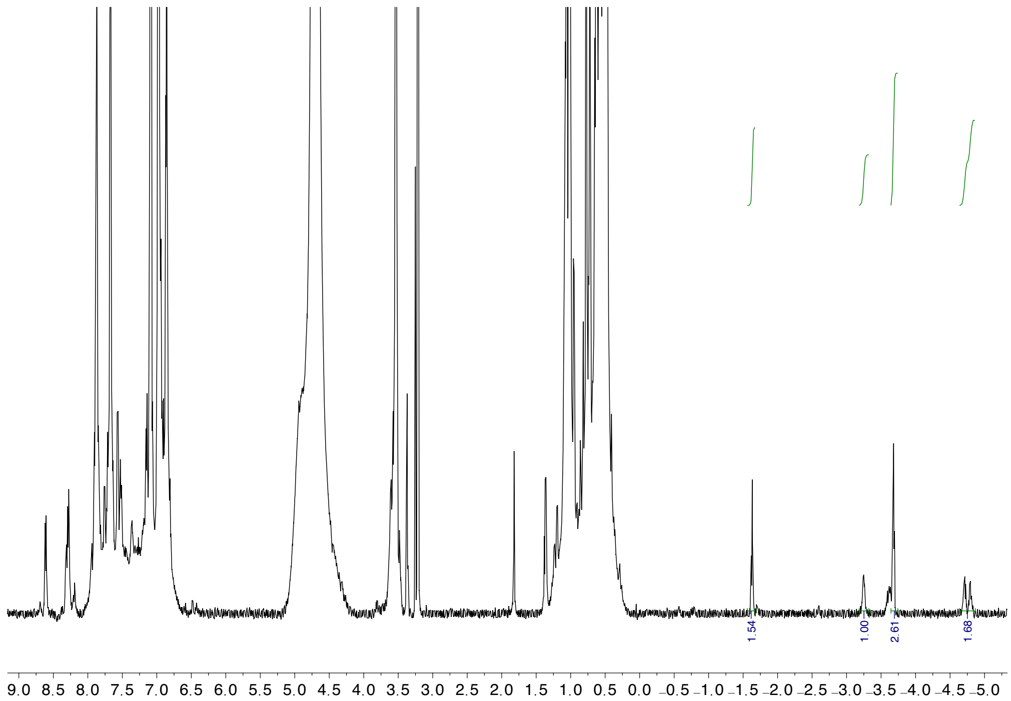

Figure S3. ^{1}H NMR spectrum of the equimolar $SiEt_4$:**1**:**2** competition mixture. Encapsulated guest peaks are integrated for clarity, with the more upfield signals being guest bound to host **2**.

Procedures and Conditions for Ketone Reduction Conditions

General Procedure A for Racemic Ketone Reductions

To a reaction tube was added the ketone substrate (1.0 mmol, 1 equiv.) and methanol (2.5 mL), which was then left to stir while cooling to 0ºC in an ice bath. Then, $NaBH_4$ (150 mg, 2.0 equiv.) was added as a solid. The mixture was left to warm to room temperature and stir overnight (~ 16 hours). The reaction was then quenched with H_2O (2 mL), and the product was extracted with EtOAc (3 x 3 mL). The combined organic layer was dried over $MgSO_4$ and concentrated in vacuo, affording the racemic alcohol product.

General Procedure B for Host-Catalyzed Ketone Reductions

To an NMR tube was added the host catalyst **1** or **2** (1.2 µmol, 0.035 equiv.) from a stock solution of premeasured concentration (5-10 mM) and diluted with pD 8.5 D_2O buffer 50 mM K_3PO_4, and CD_3OD to a total volume of 540 ul and 95 µL, respectively. Ketone (35 µmol, 1 equiv.) and pyridine borane (7 µL, 2 equiv.) were added sequentially via Hamilton syringe. The tube was capped, vigorously shaken, and quickly placed in an oil bath at 35 ºC. To stop the reaction, PEt_4Br (37 µL, 1.8 µmol, 0.053 equiv.) was added as a 50 mM stock solution in D_2O. Internal standard (3-(Trimethylsilyl)-1-propanesulfonic acid sodium salt) (20 µL, 5 µmol) was added as a 0.25 M solution in D_2O, followed by CD_3OD (50 µL) for ^{1}H NMR yield determination. The reaction mixture was then transferred

to a 2-dram vial along with excess NaCl until precipitation persisted. The product was then extracted with DCM (3 x 3 mL), and the combined organic layers were concentrated in vacuo. The product was purified by preparatory TLC in 97:3 DCM/MeOH, and isolated for chiral HPLC analysis.

General Procedure C for Racemic Ketone Functionalization

Racemic alcohol (0.11 mmol, 1 equiv.) was added to a 1-dram vial with stir bar and dissolved in DCM (0.77 mL). DMAP (0.32 mmol, 40 mg, 3.0 equiv.), NEt_3 (0.65 mmol, 90 μL, 6.0 equiv.), and BzCl (0.32 mmol, 38 μL, 3.0 equiv.) were added to the stirring vial. The mixture was stirred at room temperature for at least 3 hr after which it was concentrated in vacuo. The product was purified by preparatory TLC in hexanes, and isolated for chiral HPLC analysis.

General Procedure D for Enantioenriched Ketone Functionalization

To the crude reaction mixture isolated according to general procedure B (0.035 mmol, 1 equiv.) was added DMAP (0.11 mmol, 13 mg, 3.0 equiv.) as a stock solution in DCM (100 mg/mL, 128 μL), NEt_3 (0.21 mmol, 30 μL, 6.0 equiv.), and BzCl (0.11 mmol, 12 μL, 3.0 equiv.) as a stock solution in DCM (0.96 M, 122 μL) were added to the stirring vial. The mixture was stirred at room temperature overnight after which it was concentrated in vacuo. The product was purified by preparatory TLC in hexanes, and isolated for chiral HPLC analysis.

Representative NMR Spectra

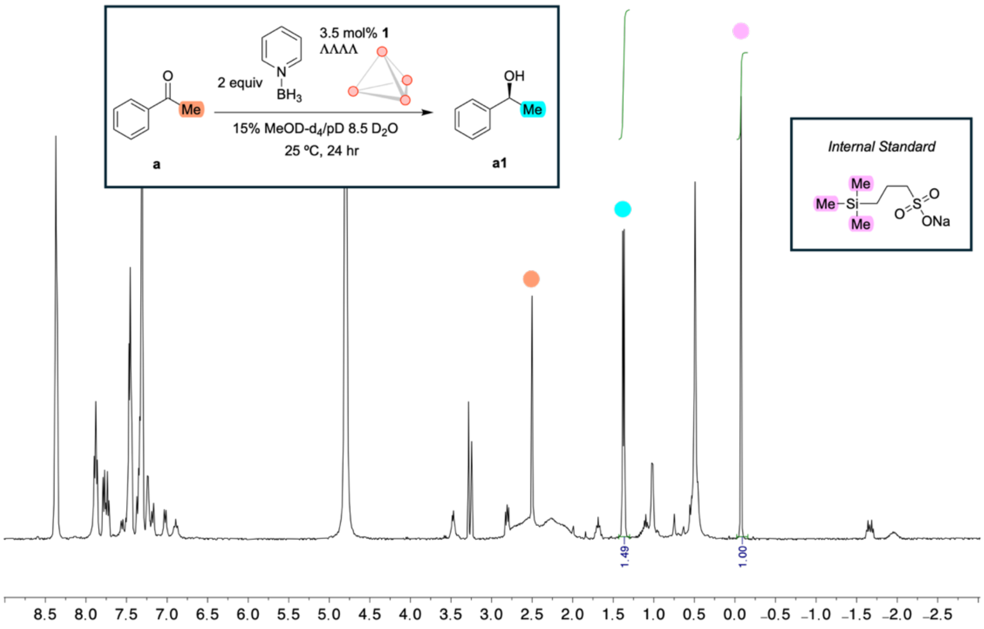

Figure S4. Representative crude ^{1}H NMR spectrum deuterated methanol/water reaction mixture. Spectrum acquired after the addition of a catalyst inhibitor (PEt_4Br) and an internal standard for yield determination. A D1 = 15 s was used for quantitative integrations.

To probe whether the pyridine borane complex is water stable under reaction conditions, an NMR monitoring experiment was conducted using a modified version of General Procedure B. Host **1** and pyridine borane was added to an NMR tube without any ketone. After heating at 35 ºC for 20 hours, no detectable decomposition of the pyridine borane was observed, suggesting the reductant is hydrolytically stable throughout the course of a typical reaction.

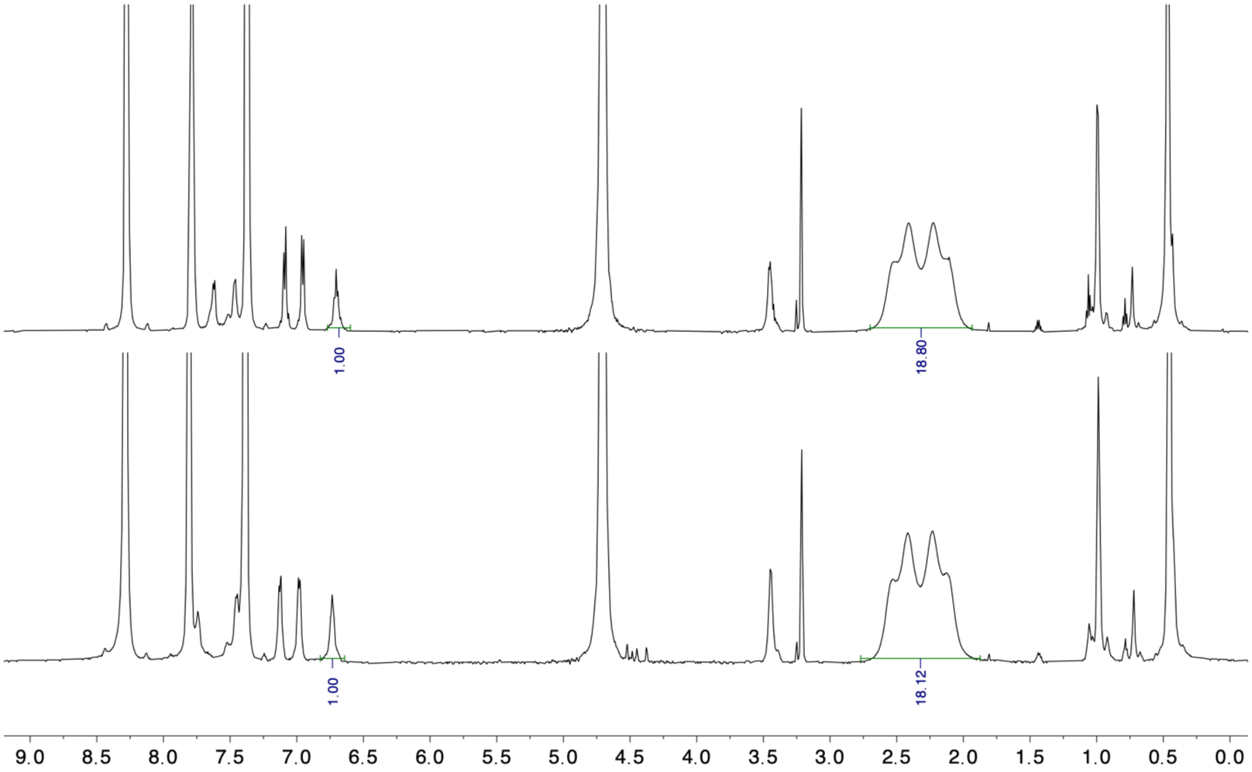

Figure S5. ^{1}H NMR spectrum of pyridine borane complex and host **1** in deuterated methanol/water reaction mixture. Spectrum acquired before (top) and after heating to 35 ºC for 20 hours (bottom). The pyridine borane hydride peak is integrated relative to a host aromatic peak in both spectra for reference.

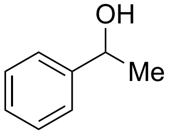

1-Phenylethanol (a1): Racemic material was used directly from a commercial bottle. Analytical data were consistent with literature reports.[2]

^{1}H NMR (500 MHz, CDCl3) δ 7.45 – 7.36 (m, 4H), 7.33 – 7.28 (m, 1H), 4.99 – 4.86 (m, 1H), 1.89 (s, 1H), 1.53 (d, *J* = 6.5 Hz, 3H).
^{13}C NMR (126 MHz, $CDCl_3$) δ 145.94, 128.64, 127.61, 125.52, 70.55, 25.29.

ChiralCel IB column (95:5 hexanes/^{i}PrOH, 1 mL/min), t_r = 7.44 min (major), t_r = 8.01 min (minor).

Enantioenriched material was prepared from **a** with hosts **1** and **2** according to General Procedure B. The reaction was stopped with PEt_4Br after 24 h and analyzed by ^{1}H NMR (quant. NMR yield for both **1** and **2**). The product was purified by prep TLC and isolated as a clear oil for HPLC analysis (14% and 84% ee for **1** and **2** respectively).

Note: Using the above HPLC conditions, the *S* alcohol from a commercial bottle was run and its single peak matched the earlier t_r, allowing for absolute stereochemistry of the major host-catalyzed product to be determined.

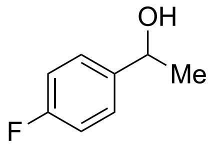

1-(4-Fluorophenyl)ethanol (b1): Racemic material was used directly from a commercial bottle. Analytical data were consistent with literature reports.[2]

^{1}H NMR (500 MHz, $CDCl_3$) δ 7.34 (ddd, *J* = 7.2, 3.8, 1.7 Hz, 2H), 7.07 – 6.95 (m, 2H), 4.92 – 4.84 (m, 1H), 1.53 – 1.44 (m, 3H).
^{13}C NMR (126 MHz, $CDCl_3$) δ 163.23, 161.28, 141.66, 141.64, 127.21, 127.14, 115.48, 115.31, 69.92, 25.43.
^{19}F NMR (471 MHz, $CDCl_3$) δ -115.34, -115.35.

ChiralCel IG column (97:3 hexanes/^{i}PrOH, 1 mL/min), t_r 12.3 min (minor, with **2**), t_r = 12.9 min (major).

Enantioenriched material was prepared from **b** with hosts **1** and **2** according to General Procedure B. The reaction was stopped with PEt_4Br after 22 h and analyzed by ^{1}H NMR (66% and quant. NMR yield for **1** and **2** respectively). The product was purified by prep TLC and isolated as a clear oil for HPLC analysis (–46% and 86% ee for **1** and **2** respectively).

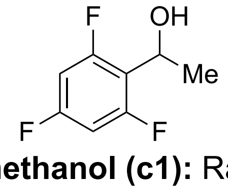

2,4,6-Trifluoro-α-methylbenzenemethanol (c1): Racemic material was prepared from **c** according to General Procedure A. (56% yield) Analytical data were consistent with literature reports.[3]

^{1}H NMR (500 MHz, $CDCl_3$) δ 6.64 (s, 2H), 5.20 (q, *J* = 6.8 Hz, 1H), 2.04 (s, 1H), 1.61 (d, *J* = 6.8 Hz, 3H).

^{13}C NMR (126 MHz, $CDCl_3$) δ 162.93, 162.22, 162.13, 162.10, 162.01, 160.95, 160.24, 160.15, 160.12, 160.03, 117.20, 117.16, 117.07, 117.03, 116.93, 116.89, 100.94, 100.74, 100.73, 100.71, 100.69, 100.51, 100.49, 62.26, 62.23, 23.50.
^{19}F NMR (471 MHz, $CDCl_3$) δ -109.24 (p, J = 7.7 Hz), -112.73 (t, J = 7.3 Hz).

ChiralCel IG column (98:2 hexanes/^{i}PrOH, 1 mL/min), t_r = 8.05 min (minor), t_r = 8.83 min (major).

Enantioenriched material was prepared from **c** with hosts **1** and **2** according to General Procedure B. The reaction was stopped with PEt_4Br after 22 h and analyzed by ^1H NMR (26% and 84% NMR yield for **1** and **2** respectively). The product was purified by prep TLC and isolated for HPLC analysis (9% and 45% ee for **1** and **2** respectively).

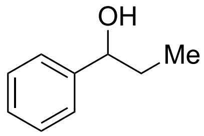

1-Phenylpropanol (d1): Racemic material was prepared from **d** according to General Procedure A. (82% yield) Analytical data were consistent with literature reports.[2]

^1H NMR (500 MHz, $CDCl_3$) δ 7.37 (d, J = 3.5 Hz, 4H), 7.33 – 7.25 (m, 1H), 4.62 (ddd, J = 7.2, 6.1, 1.9 Hz, 1H), 1.95 – 1.71 (m, 3H), 0.95 (t, J = 7.4 Hz, 3H).
^{13}C NMR (126 MHz, $CDCl_3$) δ 144.73, 128.54, 127.63, 126.10, 76.16, 32.02, 10.27.

ChiralCel IG column (99:1 hexanes/^{i}PrOH, 1 mL/min), t_r = 23.3 min (major), t_r = 24.7 min (minor).
Enantioenriched material was prepared from **d** with hosts **1** and **2** according to General Procedure B. The reaction was stopped with PEt_4Br after 22 h and analyzed by ^1H NMR (49% and 95% NMR yield for **1** and **2** respectively). The product was purified by prep TLC and isolated for HPLC analysis (50% and 62% ee for **1** and **2** respectively).

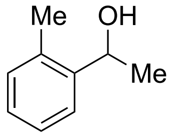

1-(2-Methylphenyl)ethanol (e1): Racemic material was prepared from **e** according to General Procedure A. (40% yield) Analytical data were consistent with literature reports.[2]

^1H NMR (500 MHz, $CDCl_3$) δ 7.43 (dd, J = 7.7, 1.4 Hz, 1H), 7.20 – 7.13 (m, 1H), 7.09 (td, J = 7.3, 1.5 Hz, 1H), 7.05 (dd, J = 7.5, 1.7 Hz, 1H), 5.05 (q, J = 6.4 Hz, 1H), 2.27 (s, 3H), 1.69 (bs, 1H), 1.39 (d, J = 6.4 Hz, 3H).

^{13}C NMR (126 MHz, $CDCl_3$) δ 143.96, 134.35, 130.50, 127.30, 126.50, 124.59, 66.95, 24.06, 19.03.

ChiralCel AD-H column (97:3 hexanes/^{i}PrOH, 1 mL/min), t_r = 9.14 min (major), t_r = 10.2 min (minor).

Enantioenriched material was prepared from **e** with hosts **1** and **2** according to General Procedure B. The reaction was stopped with PEt_4Br after 22 h and analyzed by ^1H NMR (79% and 79% NMR yield for **1** and **2** respectively). The product was purified by prep TLC and isolated for HPLC analysis (49% and 41% ee for **1** and **2** respectively).

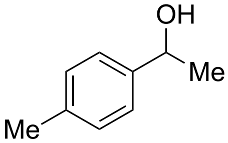

1-(4-Methylphenyl)ethanol (f1): Racemic material was used directly from a commercial bottle. Analytical data were consistent with literature reports.[2]

^1H NMR (500 MHz, $CDCl_3$) δ 7.29 (d, *J* = 7.9 Hz, 2H), 7.19 (d, *J* = 7.8 Hz, 2H), 4.89 (q, *J* = 6.4 Hz, 1H), 2.37 (s, 3H), 1.84 (bs, 1H), 1.51 (d, *J* = 6.4 Hz, 3H).
^{13}C NMR (126 MHz, $CDCl_3$) δ 142.99, 137.28, 129.29, 125.48, 70.38, 25.20, 21.21.

ChiralCel OJ-H column (95:5 hexanes/^{i}PrOH, 1 mL/min), t_r = 8.70 min (minor with **2**), t_r = 9.59 min (major).

Enantioenriched material was prepared from **f** with hosts **1** and **2** according to General Procedure B. The reaction was stopped with PEt_4Br after 22 h and analyzed by ^1H NMR (65% and quant. NMR yield for **1** and **2** respectively). The product was purified by prep TLC and isolated for HPLC analysis (–57% and 33% ee for **1** and **2** respectively).

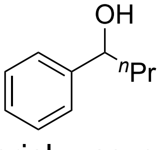

1-Phenylbutanol (g1): Racemic material was prepared from **g** according to General Procedure A. (100% yield) Analytical data were consistent with literature reports.[2]

^1H NMR (500 MHz, CDCl3) δ 7.35 (m, 4H), 7.31 – 7.25 (m, 1H), 4.68 (dd, *J* = 7.6, 5.7 Hz, 1H), 1.80 (m, 2H), 1.69 (m, 1H), 1.44 (m, 1H), 1.33 (m, 1H), 0.93 (t, *J* = 7.4 Hz, 3H).
^{13}C NMR (126 MHz, CDCl3) δ 145.07, 128.56, 127.62, 126.03, 74.57, 41.38, 19.18, 14.10.

ChiralCel IC column (98:2 hexanes/^{i}PrOH, 0.8 mL/min), t_r = 10.2 min (major), t_r = 10.6 min (minor).

Enantioenriched material was prepared from **g** with hosts **1** and **2** according to General Procedure B. The reaction was stopped with PEt_4Br after 24 h and analyzed by ^{1}H NMR (5% and 23% NMR yield for **1** and **2** respectively). The product was purified by prep TLC and isolated for HPLC analysis (12% and 41% ee for **1** and **2** respectively).

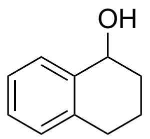

1,2,3,4-Tetrahydronaphthalen-1-ol (h1): Racemic material was used directly from a commercial bottle. Analytical data were consistent with literature reports.[2]

^{1}H NMR (500 MHz, CDCl3) δ 7.46 – 7.41 (m, 1H), 7.24 – 7.18 (m, 2H), 7.13 – 7.09 (m, 1H), 4.79 (dd, *J* = 5.8, 3.9 Hz, 1H), 2.84 (dt, *J* = 16.5, 5.4 Hz, 1H), 2.73 (m, 1H), 2.06 – 1.86 (m, 3H), 1.79 (m, 1H), 1.72 (s, 1H).
^{13}C NMR (126 MHz, CDCl3) δ 138.93, 137.25, 129.15, 128.78, 127.72, 126.31, 68.29, 32.41, 29.37, 18.92.

ChiralCel AD-H column (99:1 hexanes/^{i}PrOH, 1 mL/min), t_r = 21.4 min (minor, with **2**), t_r = 24.8 min (major).

Enantioenriched material was prepared from **h** with hosts **1** and **2** according to General Procedure B. The reaction was stopped with PEt_4Br after 22 h and analyzed by ^{1}H NMR (quant. NMR yield for both **1** and **2**). The product was purified by prep TLC and isolated for HPLC analysis (–81% and 64% ee for **1** and **2** respectively).

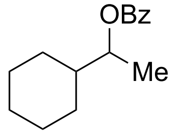

1-Cyclohexylethyl benzoate (i1): Racemic material was prepared from **i** according to a modified version of General Procedure A. After extraction, the crude material was subjected to General procedure D, affording the functionalized product (65% yield) Analytical data were consistent with literature reports.[4]

^{1}H NMR (500 MHz, CDCl3) δ 8.05 (d, *J* = 8.4 Hz, 2H), 7.54 (tt, *J* = 7.5, 1.3 Hz, 1H), 7.44 (t, *J* = 7.8 Hz, 2H), 5.00 (p, *J* = 6.3 Hz, 1H), 1.94 – 1.64 (m, 5H), 1.60 (m, 1H), 1.30 (d, *J* = 6.4 Hz, 3H), 1.29 – 1.03 (m, 5H).
^{13}C NMR (126 MHz, CDCl3) δ 166.33, 132.80, 131.12, 129.66, 128.42, 75.41, 42.94, 28.84, 28.60, 26.57, 26.24, 26.20, 17.29.

ChiralCel IG column (100% hexanes, 0.5 mL/min), t_r = 9.1 min (major), t_r = 9.5 min (minor).

Enantioenriched material was prepared from **i** with hosts **1** and **2** according to a modified version of General Procedure B. The reaction was stopped with PEt_4Br after 22 h and analyzed by ^1H NMR (0% and quant. NMR yield for **1** and **2** respectively). After extraction, the crude material was then functionalized with General Procedure D, and the product was purified by prep TLC and isolated for HPLC analysis (86% ee for **2**).

Ketone Reduction Kinetics and Mechanistic Investigation

General Procedure for Kinetics Experiments
To an NMR tube was added host **1** or **2** (0.59 – 2.35 mM) as a pre-made stock solution in MeOD-d_4 /D_2O, followed by sodium 4-fluorobenzoate as internal standard (0.01 mmol, 16.5 mM) as a pre-made stock solution (0.15 M) in D_2O. Additional amounts of MeOD-d_4 and 60 mM K_3PO_4 pD 8 D_2O buffer were added to dilute the reaction to the appropriate concentration as needed. Ketone **b** (0.01 mmol, 16.5 mM) was added to the NMR tube as a pre-made stock solution in MeOD-d_4. Pyridine borane (3.0 μL) was added via Hamilton syringe; the tube was shaken and inverted twice before being immediately inserted into the NMR spectrometer. Acquisition was started within 2 minutes of reductant addition.

Notes: An identical sample was used to tune and shim the spectrometer probe before the reaction run(s). Ketone and internal standard stock solutions, and pyridine borane were stored in vials with a Teflon cap and sealed with electrical tape in a -20°C freezer to prolong stability. Host stock solutions were stored in vials with Teflon cap and sealed with electrical taped at room temperature in an air-free wet glovebox.

Acquisition parameters:

o1p = -111; sw = 25; d1 = 1; ns = 1; acq. = 1s with fixed 3-10 s delay between scans for host **2** and 30-60 s for host **1**

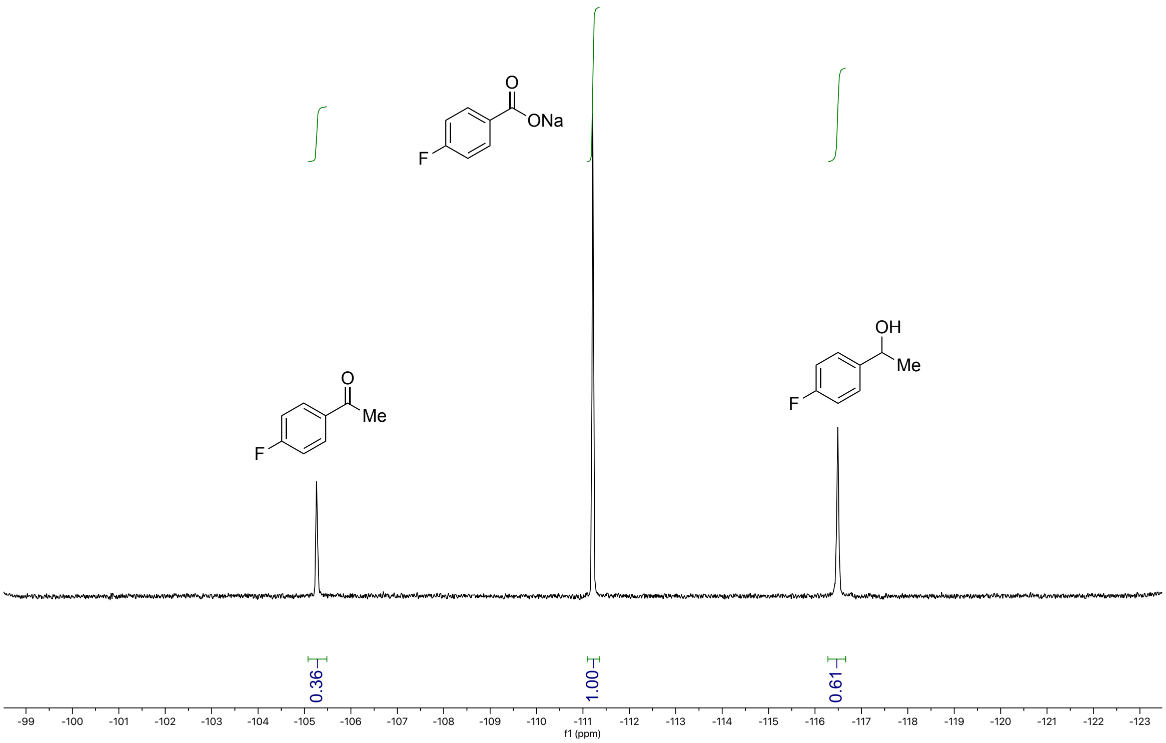

Figure S6. Representative ^{19}F NMR spectrum showing internal standard, starting material, and product peaks.

Example Reaction Profiles

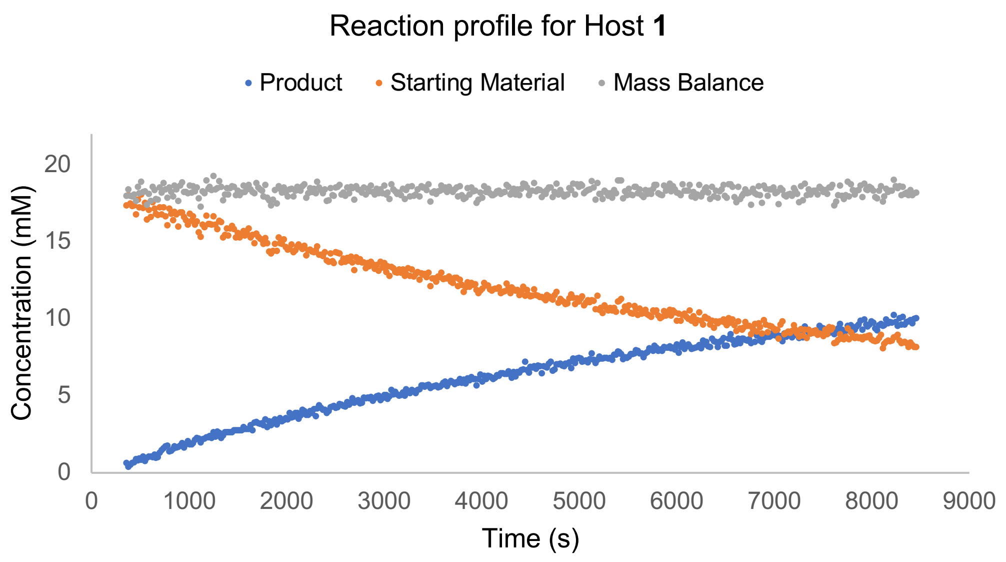

Figure S7. Representative reaction profile of the host **1**-catalyzed reduction of **b**.

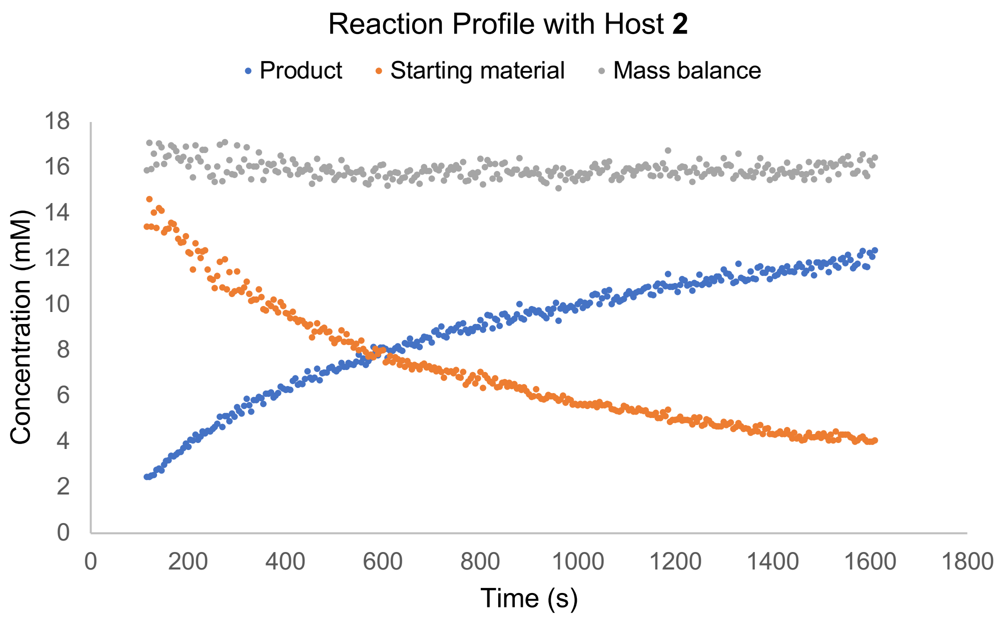

Figure S8. Representative reaction profile of the host **2**-catalyzed reduction of **b**.

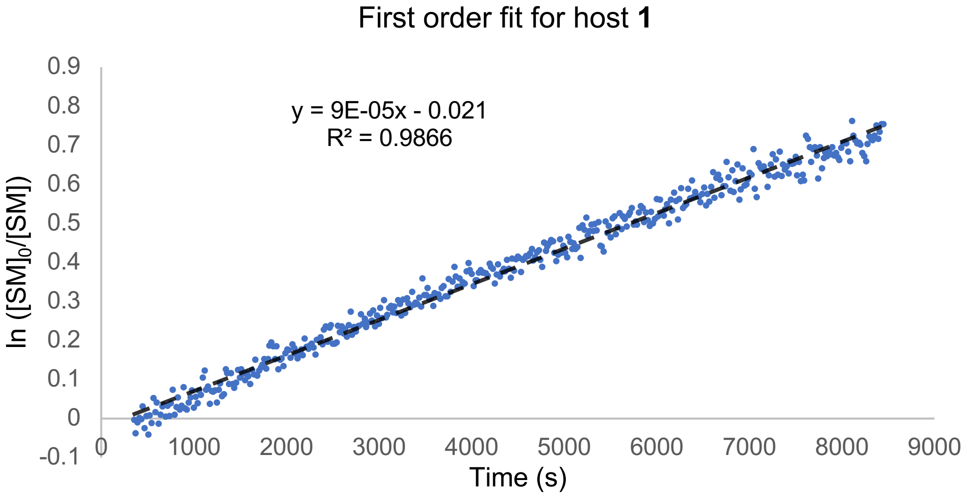

Figure S9. Representative kinetic data for the host **1**-catalyzed reduction of **b** fit to first-order in ketone (SM).

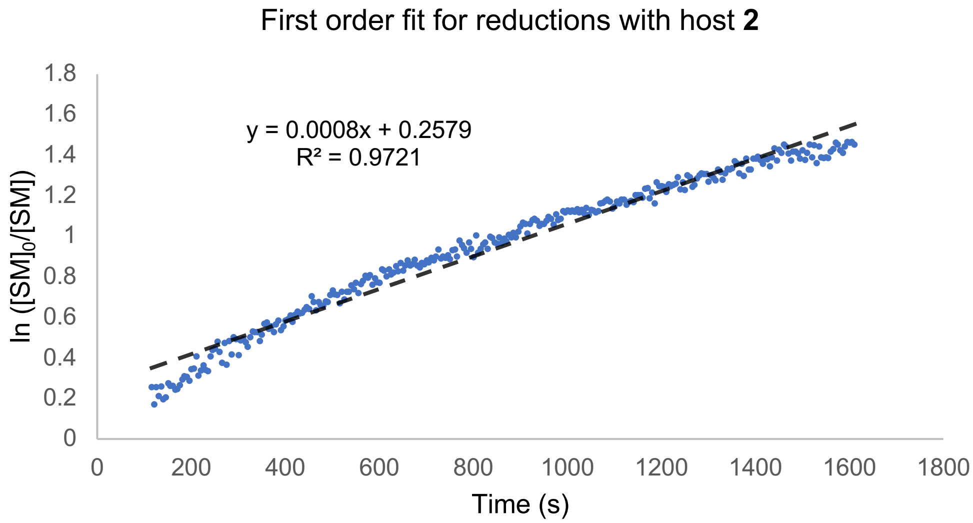

Figure S10. Representative kinetic data for the host **2**-catalyzed reduction of **b** fit to first-order in ketone (SM).

Extracted Rate Constants

	Temp (K)	k_{obs} (s^{-1})	[cat] (mM)	k_{obs}/[cat] (s^{-1} mM^{-1})	Average k_{obs} (s^{-1} mM^{-1})	Error (s^{-1} mM^{-1})
run 1-BH_3	298	0.00088	0.59	0.0015	1.4E-03	6.3E-05
run 2-BH_3	298	0.00085	0.59	0.0014		
run 3-BH3	298	0.00080	0.59	0.0014		
run 1-BH_3	308	0.00088	0.31	0.0029	2.9E-03	7.6E-05
run 2-BH_3	308	0.00085	0.31	0.0028		
run 3-BH_3	308	0.00090	0.31	0.0029		
run 1-BH_3	318	0.00108	0.31	0.0035	4.3E-03	8.5E-04
run 2-BH_3	318	0.00129	0.31	0.0042		
run 3-BH_3	318	0.00160	0.31	0.0052		
run 1-BH_3	328	0.00141	0.31	0.0046	4.6E-03	6.8E-06
run 2-BH_3	328	0.00140	0.31	0.0046		
run 1-BD_3	298	0.00042	0.59	0.0007	7.4E-04	5.0E-05
run 2-BD_3	298	0.00042	0.59	0.0007		
run 3-BD_3	298	0.00047	0.59	0.0008		

Table S1: Extracted rate constants (k_{obs}) for the host **2**-catalyzed reduction of **b** in replicate at different temperatures. The "BD_3" runs used deuterated pyridine borane for KIE analysis.

	Temp (K)	k_{obs} (s^{-1})	Average k_{obs} (s^{-1} mM^{-1})	Error (s^{-1})
run 1-BH3	298	1.12E-08	1.1E-08	6.08E-07
run 2-BH3	298	1.01E-08		
run 3-BH3	298	1.03E-08		

Table S2: Extracted rate constants (k_{obs}) for the background reduction of **b** in the absence of any host catalyst in replicated.

	Temp (K)	k_{obs} (s^{-1})	[cat] (mM)	k_{obs}/[cat] (s^{-1} mM^{-1})	Average k_{obs} (s^{-1} mM^{-1})	Error (s^{-1} mM^{-1})
run 1-BH3	298	1.79E-05	1.2	1.5E-05		
run 2-BH3	298	1.73E-05	1.2	1.5E-05	1.5E-05	6.1E-07
run 3-BH3	298	1.87E-05	1.2	1.6E-05		
run 1-BH3	318	1.46E-04	2.4	6.2E-05		
run 2-BH3	318	1.03E-04	2.4	4.4E-05	4.8E-05	1.2E-05
run 3-BH3	318	9.12E-05	2.4	3.9E-05		
run 1-BH3	328	2.20E-04	2.4	9.4E-05		
run 2-BH3	328	1.76E-04	2.4	7.5E-05	7.7E-05	1.6E-05
run 3-BH3	328	1.46E-04	2.4	6.2E-05		
run 1-BH3	338	4.75E-04	2.4	2.0E-04		
run 2-BH3	338	4.19E-04	2.4	1.8E-04	1.9E-04	1.4E-05
run 3-BH3	338	3.39E-04	1.9	1.8E-04		
run 1-BD3	338	1.52E-04	2.4	6.5E-05		
run 2-BD3	338	1.38E-04	2.4	5.9E-05	6.0E-05	4.3E-06
run 3-BD3	338	1.32E-04	2.4	5.6E-05		

Table S3: Extracted rate constants (k_{obs}) for the host **1**-catalyzed reduction of **b** in replicate at different temperatures. The "BD_3" runs used deuterated pyridine borane for KIE analysis.

Mechanistic Discussion of k_{obs}

Given that hydride delivery is rate-limiting with both hosts, all proceeding steps have some contribution to k_{obs} and, by extension, to the activation enthalpies and entropies extracted from Eyring analyses. Saturation kinetics observed with pyridine borane allows us to assume all host present is bound to the cofactor.

$$\mathrm{H + S} \underset{k_{-1}}{\overset{k_1}{\rightleftharpoons}} \mathrm{HS} \xrightarrow{k_2} \mathrm{P}$$

For this discussion, we consider pre-equilibrium of ketone (S) binding to the pyridine borane host complex (H), followed by hydride-delivery within HS to afford products (P). For simplicity, we assume that the ketone gets protonated upon binding and that HS contains the protonated substrate (concerted binding and protonation). It is reasonable to assume that product release is not rate-determining; we hypothesize turnover is facilitated by the increased water solubility of the alcohol relative to the ketone, making it a weaker binding guest.

Under the preequilibrium approximation, consistent with the reversible nature of ketone protonation (i.e. deprotonation is faster than hydride delivery), the following rate law is obtained, where $K_D = k_1/k_{-1}$ (the dissociation constant of the HS complex):

$$r = \frac{k_2[H]_{tot}[S]}{K_D} = k_{obs}[S]$$

The catalytic efficiency is thus dependent on the rate of hydride-delivery as well as the extent to which the cavity favors substrate binding. Notably, we anticipate that differences in K_D's between hosts **1** and **2** are minimal, particularly given the small (1:2 and 1:5; See Figure 4D) differences in binding affinities for screened neutral and cationic guests. Therefore, we propose that 100-fold differences in rate constants (at 298K) stem mainly from differences in rate-limiting hydride-delivery (k_2).

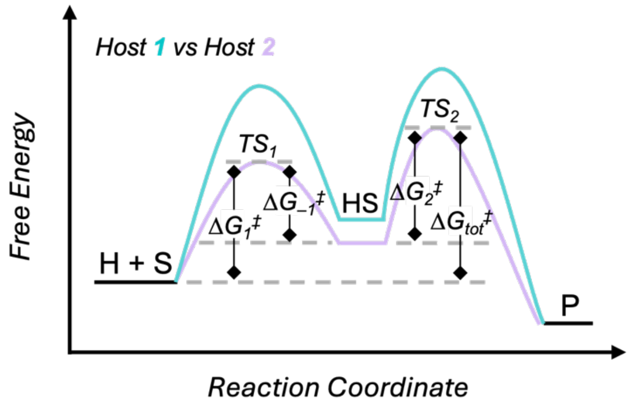

Figure S11. A qualitative reaction coordinate diagram relevant intermediates and their associated activation Free energies for host catalysts **1** and **2**.

Converting k_{obs} to $\Delta G_{tot}^‡$ and expressing it in terms of the activation free energies for each step, we get that $\Delta G_{tot}^‡ = \Delta G_1^‡ - \Delta G_{-1}^‡ + \Delta G_2^‡$ as illustrated in the reaction coordinate diagram above. The measured k_{obs} and its corresponding $\Delta G_{tot}^‡$ describe the energy difference between an intermediate containing neutral substrate and cofactor and the highest transition state (TS_2) consisting of a cationic ketone-pyridine borane complex. As a result, when comparing the differences in rates between hosts **1** and **2**, it is reasonable to assume that the reduced activation enthalpy with **2** stems from preferential stabilization of a transition state with cationic substrates over ground states in which they are neutral. This mechanistic analysis in addition to experimental data supporting the stronger affinity of **2** over **1** for cations supports enhanced cation–π interactions in the former leading to better rate enhancements.

Eyring Analysis on Enantioselectivity

General procedure for Eyring Analysis Reductions

General procedure B was followed with the ketones of interest (**a–d**). Reactions were run at temperatures ranging from 25ºC – 65ºC and were left until > 50% conversion was achieved (varies for each substrate, catalyst, and temperature combination, most were run ~24 h). Enantiomeric excess was determined by chiral **HPLC** analysis using conditions previously specified for each respective product (see scope section). The differential activation parameters were then extracted by fitting the data to R ln(er) = $-\Delta\Delta H^{‡}/T + \Delta\Delta S^{‡}$ where R = 1.986 cal/mol•K.

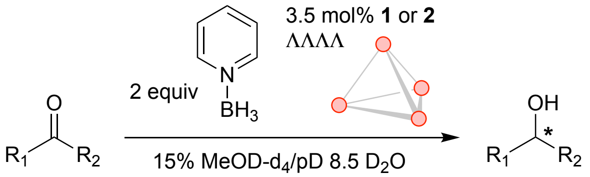

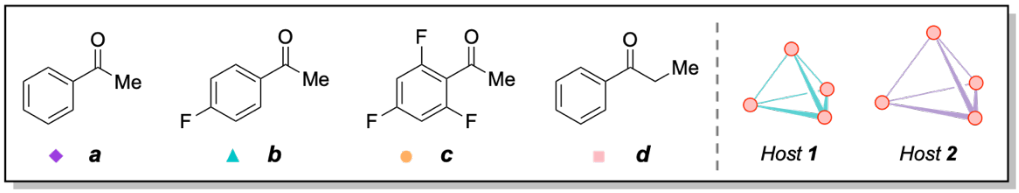

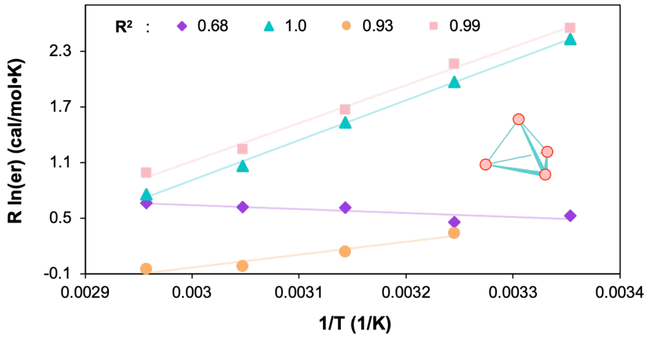

1		ΔΔH‡ (kcal/mol)	ΔΔS‡ (cal/mol•K)
◆	a	0.44 ± 0.2	2.0 ± 0.6
▲	b	– 4.3 ± 0.1	– 12 ± 0.3
●	c	– 1.4 ± 0.3	– 4.2 ± 0.8
■	d	– 4.1 ± 0.2	– 11 ± 0.6

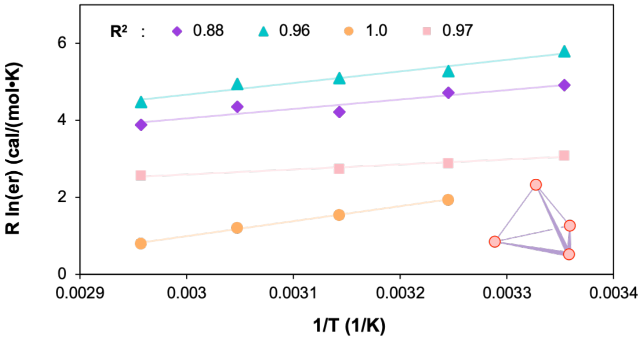

2		ΔΔH‡ (kcal/mol)	ΔΔS‡ (cal/mol•K)
◆	a	– 2.4 ± 0.6	– 3.3 ± 1.6
▲	b	– 3.0 ± 0.4	– 4.4 ± 1.2
●	c	– 3.9 ± 0.1	– 11 ± 0.4
■	d	– 1.3 ± 0.2	– 1.3 ± 0.5

Figure S12. Summarized data for Eyring analysis on selectivity in the reduction of ketones **a–d** with catalysts **1** and **2**.

Reported errors were from standard error in the slope and intercept for $\Delta\Delta H^{\ddagger}$ and $\Delta\Delta S^{\ddagger}$, respectively, obtained from the linear regression. Notably, despite certain substrates giving opposite enantiomer products between host catalysts, all were % ee values were plotted as positive unless the major enantiomer achieved with a given substrate and catalysts switched with temperature.

	**% ee with Host 1**			
**T (ºC)**	**a**	**b**	**c**	**d**
25	13.2	54.6	–	52.3
35	11.5	45.9	8.6	49.6
45	15.4	36.8	3.6	39.7
55	15.4	26.2	– 0.5	30.3
65	16.6	18.9	– 1.1	24.3

Figure S13. % ee values achieved in the reduction of ketones **a**–**d** with catalyst **1** at different temperatures.

	**% ee with Host 2**			
**T (ºC)**	**a**	**b**	**c**	**d**
25	84.5	89.8	–	65.1
35	83.0	86.9	45.2	62.2
45	78.6	85.7	36.8	59.8
55	80.0	84.7	29.3	–
65	75.3	81.0	19.8	56.9

Figure S14. % ee values achieved in the reduction of ketones **a**–**d** with catalyst **2** at different temperatures.

Note: The same procedure and data analysis as above was conducted for ketone **i**, with minor changes. The alcohol product was benzoyl-functionalized for chiral HPLC analysis. Only host **2** was used as **1** did not catalyze the reduction.

T (ºC)	%ee with 2
25	84.8
35	83.9
45	81.9
55	80.0
65	75.8

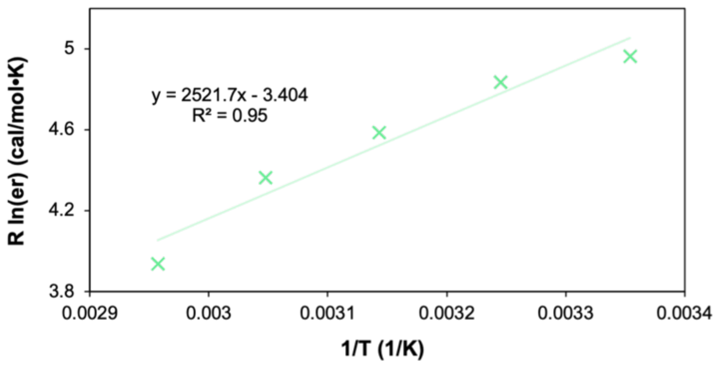

$\Delta\Delta H^{\ddagger}$ = – 2.5 ± 0.4 kcal/mol $\Delta\Delta S^{\ddagger}$ = – 3.4 ± 1.1 cal/mol•K

Figure S15. % ee at different temperatures, associated Eyring plot, and the extracted kinetic parameters for the reduction of ketone **i** with catalyst **2**.

Differential Enthalpy–Entropy Compensation

Plots of $\Delta\Delta H^{\ddagger}$ as a function of $\Delta\Delta S^{\ddagger}$ for each substrate are shown below, separated by host, displaying strong linear correlations. The symbols for each data point are consistent with the notation for each relevant substrate shown above.

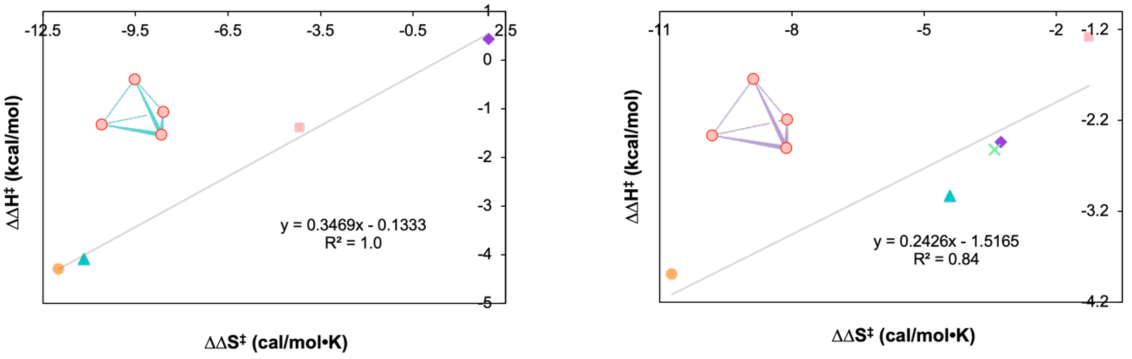

Figure S16. Different enthalpy–entropy plots for hosts **1** (left) and **2** (right). Each data point corresponds to the differential enthalpy and entropy extracted via Eyring analysis for the reduction for ketones **a**–**d**. The graph for host **2** also includes ketone **i** as a data point.

Eyring Analysis on Rates

The Eyring equation was used to extrapolate the activation parameters of host-mediated ketone reductions:

$$k = \frac{\kappa k_B T}{h} e^{\frac{\Delta S^{\ddagger}}{R}} e^{\frac{-\Delta H^{\ddagger}}{RT}};$$

$$\ln\frac{k}{T} = \frac{-\Delta H^{\ddagger}}{R} \cdot \frac{1}{T} + \ln\frac{\kappa k_B}{h} + \frac{\Delta S^{\ddagger}}{R};$$

$$\text{where k} = \text{reaction rate constant;}$$
$$\kappa = \text{transmission coefficient; } k_B = \text{Boltzmann constant; h} = \text{Planck's constant;}$$
$$\Delta S^{\ddagger} = \text{entropy of activation; } \Delta H^{\ddagger} = \text{enthalpy of activation}$$

It is assumed that the transmission coefficient $\kappa = 1$ and that the enthalpy and entropy of activation are constant within the measured temperature range.

Temp (K)	Avg. ln(k/T)	1/T (1/K)
298	-12.244	0.003356
308	-11.580	0.003247
318	-11.215	0.003145
328	-11.173	0.003049

Table S4: Summarized data for Eyring analysis on rates for host-**2** catalyzed reduction of **b**

Temp (K)	Avg. ln(k/T)	1/T (1/K)
298	-16.787	0.003356
318	-15.722	0.003145
328	-15.280	0.003049
338	-14.418	0.002959

Table S5: Summarized data for Eyring analysis on rates for host-**1** catalyzed reduction of **b**

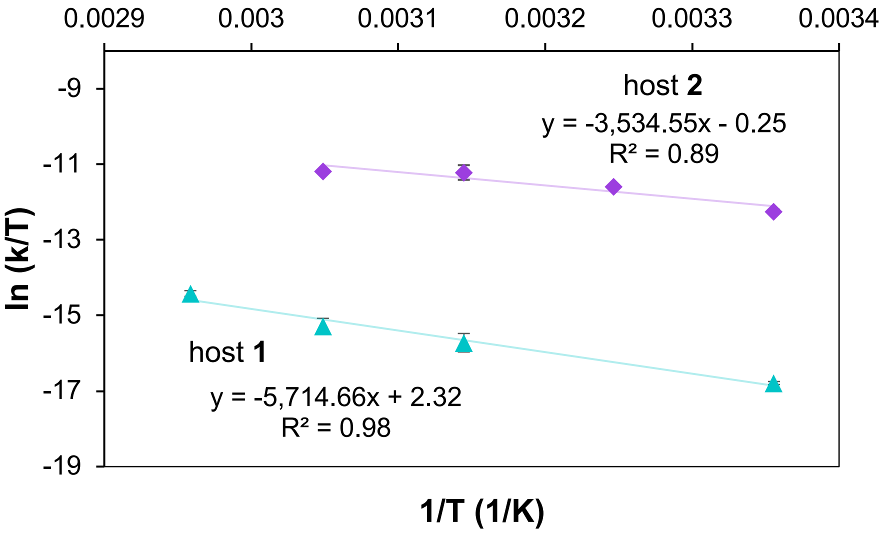

Figure S17. Eyring plots for host catalysts **1** and **2** in the reduction of ketone **b**.

NMR Spectra

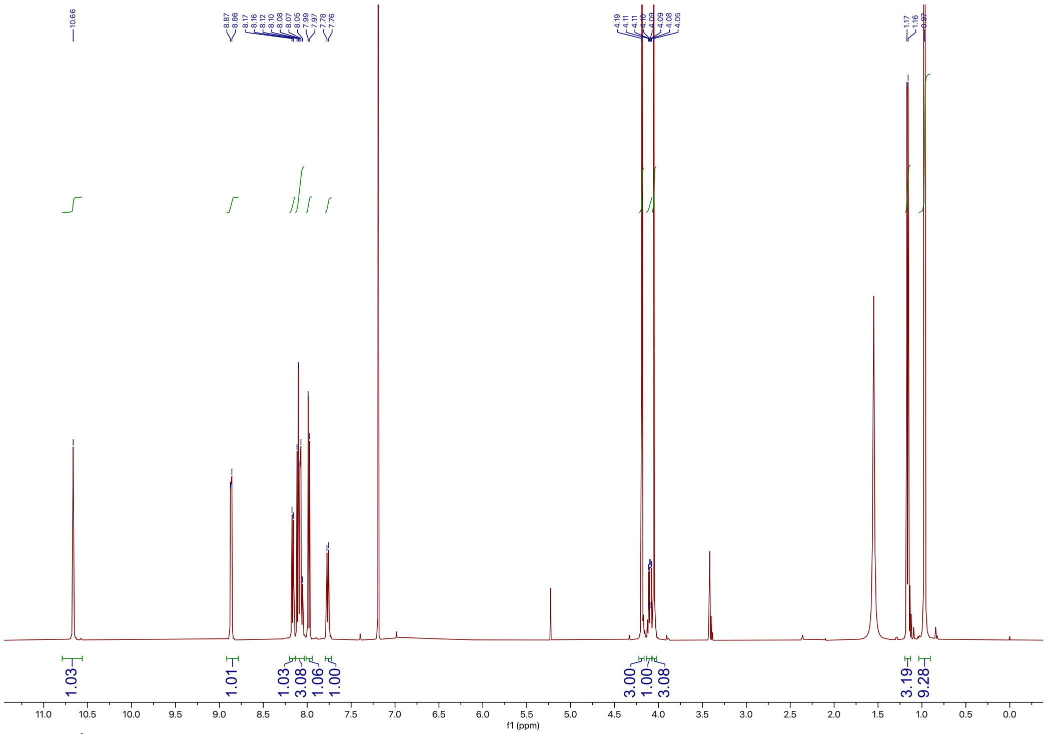

Figure S18: ^{1}H NMR spectrum of protected ligand **S2** (500 MHz, $CDCl_3$)

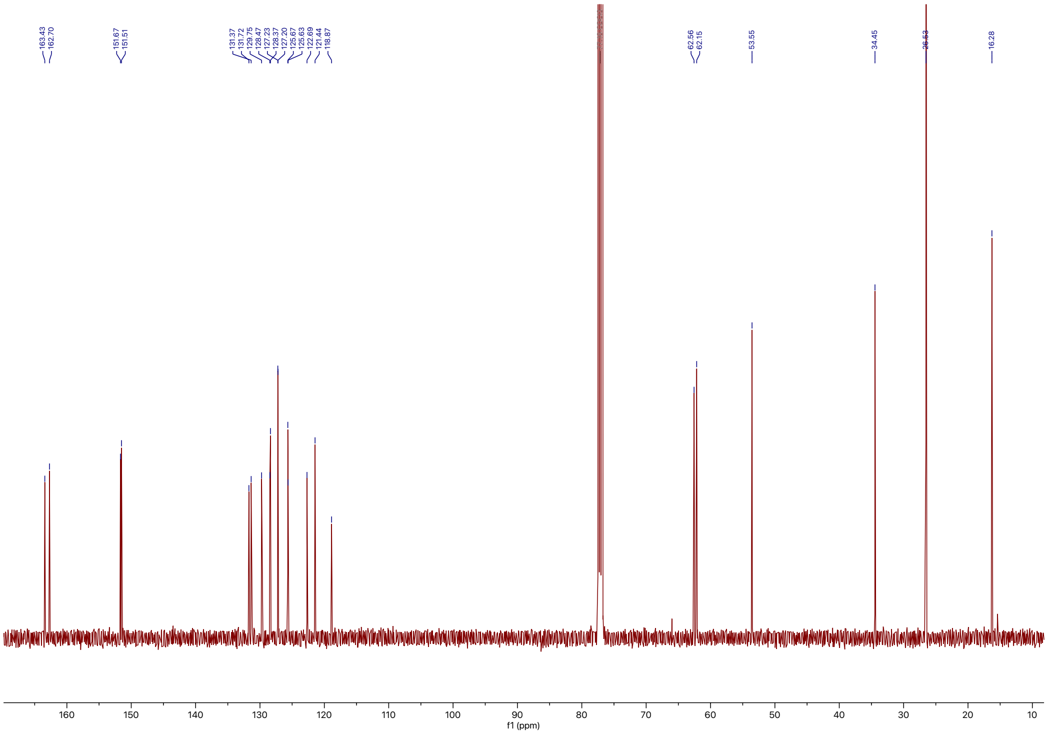

Figure S19: ^{13}C NMR spectrum of protected ligand **S2** (126 MHz, $CDCl_3$)

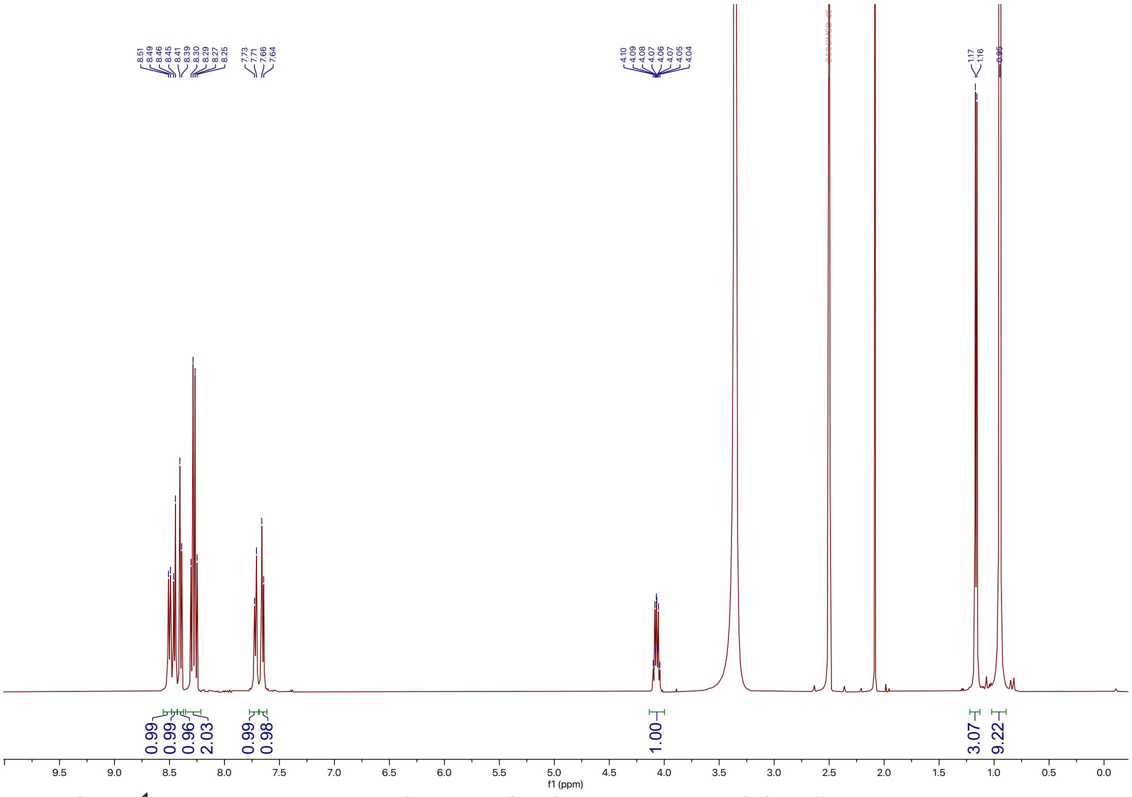

Figure S20: ^{1}H NMR spectrum of ligand **S3** (500 MHz, DMSO-d_6)

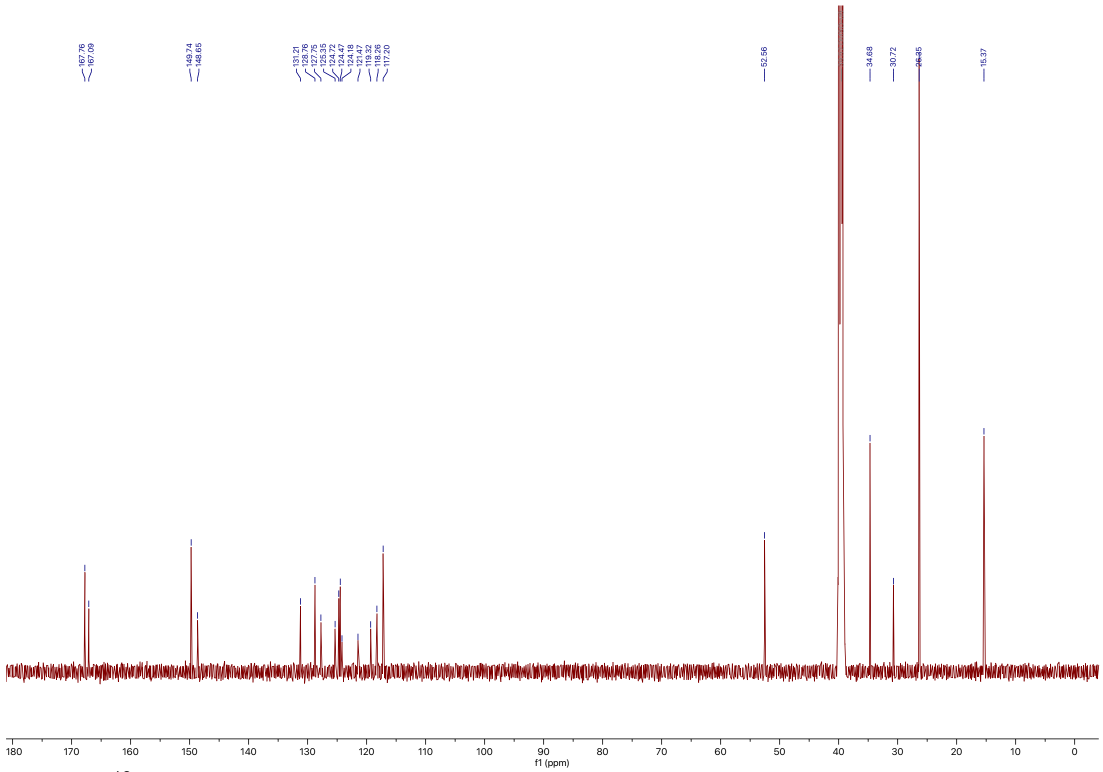

Figure S21: ^{13}C NMR spectrum of ligand **S3** (126 MHz, DMSO-d_6)

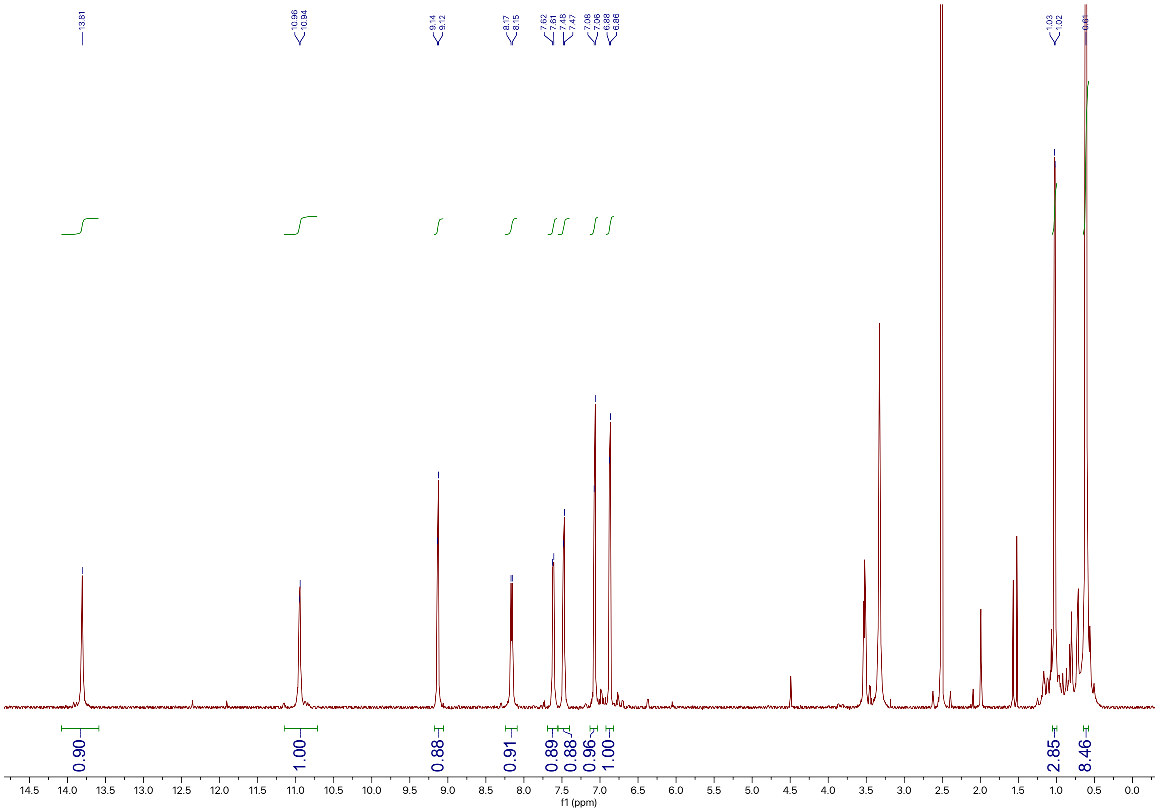

Figure S22: ^{1}H NMR spectrum of empty host **2** (500 MHz, MeOD-d_4).

Computational Workflow

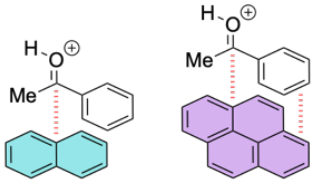

Calculations were run remotely using clusters at the Molecular Graphics and Computation Facility (MGCF) at the University of California, Berkeley (NIH S10OD034382). Density functional theory (DFT) calculations were carried out using gaussian software, all at the M06/6-31G(d,p) level of theory in the gas phase.[5] Protonated forms of ketones **a**–**d** were subjected to geometry optimization and frequency calculations, as were molecules of naphthalene and pyrene as truncated forms of the host walls. For the stacked complexes, substrates were placed parallel to the aromatic plane of the naphthalene or pyrene, and subject first to a rapid geometry optimization using a UFF force field. Using the structures obtained for acetophenone (**a**), substrate derivatizations were made such that all the adducts started from roughly the same initial coordinates before DFT optimization. These initial coordinate for Naph•••**a** and Pyr•••**a** are provided below for reference. These structures were then subjected to an optimization and frequency calculation. The enthalpies and free energies with thermal corrections (E) were extracted from each optimized file, and the interaction energies were calculated as ($\mathbf{E_{complex}} - \mathbf{E_{pyr\ or\ naph}} - \mathbf{E_{substrate\ H+}}$) • 627.9505 kcal/mol using both enthalpy and free energy values for comparison. While the actual magnitudes of the interaction energies do not have much applicable meaning to solution state reactivity given, they are gas phase calculations

lacking the complete host structure and pyridine borane cofactor, this approach is feasible for assessing trends in interaction strength for comparison with experimental data.

Interaction Energies and Correlation Data

Naphthalene Complex	Interaction Free Energy (kcal/mol)	Interaction Enthalpy (kcal/mol)	$\lvert\Delta\Delta H^{\ddagger}\rvert$ (kcal/mol)
a	–1.43	–12.93	0.44
b	–1.09	–12.92	4.3
c	–4.39	–16.44	1.4
d	0.0100	–11.80	4.1

Table S6: Computationally calculated interaction free energies and enthalpies as well as experimentally determined differential enthalpies for substrates **a**–**d** and host **1**

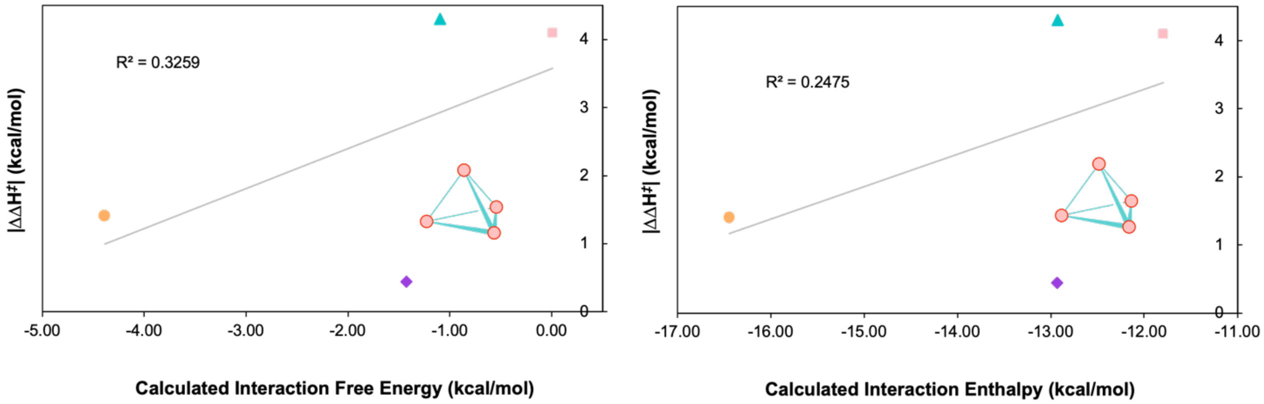

Figure S23: Correlations between different enthalpy and calculated interaction free energy and enthalpies for substrates **a**–**d** and host **1**.

Pyrene Complex	Interaction Free Energy (kcal/mol)	Interaction Enthalpy (kcal/mol)	$\lvert\Delta\Delta H^{\ddagger}\rvert$ (kcal/mol)
a	–6.55	–19.54	2.4
b	–6.80	–20.03	3
c	–7.40	–21.10	3.9
d	–5.70	–18.81	1.3

Table S7: Computationally calculated interaction free energies and enthalpies as well as experimentally determined differential enthalpies for substrates **a**–**d** and host **2**.

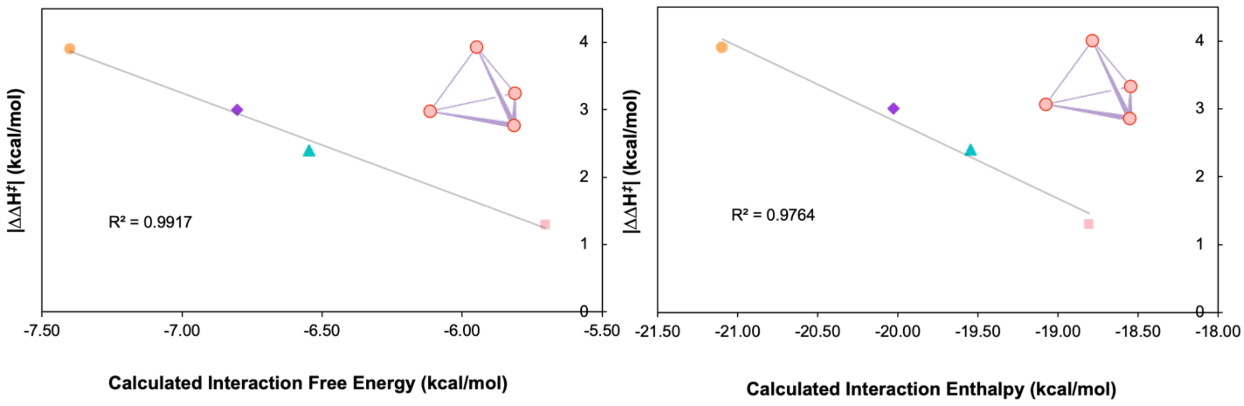

Figure S24: Correlations between different enthalpy and calculated interaction free energy and enthalpies for substrates **a–d** and host **2**.

XYZ Coordinates for Interaction Energy Calculations

Naphthalene

C	-2.423796	-0.706149	0.000016
C	-1.239224	-1.397078	0.000018
C	-2.423797	0.706148	-0.000007
C	-1.239226	1.397077	0.000015
C	-0.000001	0.712993	0.000008
C	0.000001	-0.712993	0.000003
H	-3.368575	1.244582	-0.000056
H	-1.232514	2.486349	-0.000035
C	1.239227	-1.397077	-0.000005
C	2.423797	-0.706148	0.000004
C	1.239224	1.397078	0.000003
C	2.423795	0.706149	0.00001
H	3.368575	-1.244581	-0.000018
H	1.232513	2.48635	-0.000063
H	3.368573	1.244583	-0.000043
H	1.232518	-2.486349	-0.000045
H	-1.232515	-2.48635	-0.000068
H	-3.368573	-1.244584	-0.000065

Pyrene

C	1.422886	-1.2304	-0.000013

C	0.710708	0	-0.000026
C	0.678189	-2.454485	-0.000042
C	-0.678189	-2.454485	-0.000042
C	-1.422886	-1.2304	-0.000014
C	-0.710708	0	-0.000026
H	1.23138	-3.392571	-0.000077
H	-1.23138	-3.392571	-0.000078
C	-1.422886	1.2304	-0.000013
C	-2.821082	1.206012	0.000034
C	-2.821082	-1.206012	0.000034
C	-3.510147	0	0.000061
H	-3.364324	2.1498	0.000046
H	-3.364324	-2.1498	0.000045
H	-4.597615	0	0.000107
C	2.821082	-1.206012	0.000034
C	3.510147	0	0.000061
C	2.821082	1.206012	0.000034
C	1.422886	1.2304	-0.000014
C	0.678189	2.454485	-0.000042
C	-0.678189	2.454485	-0.000041
H	3.364324	-2.1498	0.000046
H	3.364324	2.1498	0.000045
H	4.597615	0	0.000107
H	1.23138	3.392571	-0.000078
H	-1.23138	3.392571	-0.000077

*Protonated Ketone **a***

C	-0.173803	-0.031579	0.000131
C	0.489696	1.217568	-0.000049
C	1.867934	1.263257	-0.000107
C	2.599762	0.07271	-0.00006
C	1.959451	-1.169201	0.000054
C	0.581815	-1.229095	0.000155
H	-0.075586	2.145684	-0.000097
H	2.38226	2.219015	-0.000196
H	3.686014	0.114085	-0.000122
C	-1.591151	-0.085191	0.000207
H	0.071532	-2.187612	0.00023

H	2.544111	-2.083725	0.000067
O	-2.129306	-1.266649	-0.000273
C	-2.468415	1.107946	0.000095
H	-2.26843	1.727182	0.881931
H	-3.52822	0.840062	0.000203
H	-2.268564	1.726847	-0.882023
H	-3.100409	-1.246834	-0.000356

Protonated Ketone ***b***

C	-0.60437	-0.035909	0.000026
C	0.080788	1.204992	-0.000031
C	1.453965	1.2416	-0.000032
C	2.150521	0.030008	-0.000004
C	1.513417	-1.213492	0.000034
C	0.140393	-1.244395	0.000061
H	-0.469954	2.141675	-0.000058
H	2.010018	2.173416	-0.000056
F	3.457632	0.062856	-0.000014
C	-2.017601	-0.071792	0.000027
H	-0.381433	-2.196635	0.000087
H	2.113395	-2.117813	0.000045
O	-2.57111	-1.248515	-0.000101
C	-2.880932	1.132798	0.000059
H	-2.676844	1.749592	0.882597
H	-3.94351	0.876065	0.000192
H	-2.677039	1.749434	-0.882641
H	-3.541517	-1.21617	-0.000073

Protonated Ketone ***c***

C	0.541964	0.031951	-0.000099
C	-0.18022	-1.196119	-0.000159
C	-1.552612	-1.264525	-0.00003
C	-2.262628	-0.068912	0.000038
C	-1.641354	1.182284	0.000024
C	-0.275398	1.198268	-0.000029
F	0.491065	-2.326662	-0.000295
H	-2.061467	-2.222607	-0.00007
F	-3.565284	-0.112881	0.000162

C	1.969589	0.081106	0.000117
F	0.330508	2.389399	0.000054
H	-2.217185	2.101662	0.000084
O	2.617611	1.198156	-0.00026
C	2.859498	-1.095932	0.000514
H	2.666867	-1.726517	0.875232
H	3.897107	-0.760848	0.002244
H	2.66957	-1.724554	-0.876279
H	2.044597	1.990196	-0.000682

*Protonated Ketone **d***

C	-0.292584	0.08991	-0.000003
C	-0.848591	-1.209777	0.000019
C	-2.218683	-1.370955	0.000012
C	-3.048691	-0.247171	-0.000032
C	-2.514653	1.043141	-0.000056
C	-1.146178	1.218018	-0.000036
H	-0.208743	-2.088144	0.000064
H	-2.650046	-2.366982	0.000042
H	-4.127534	-0.380175	-0.000048
C	1.117936	0.26952	0.000054
H	-0.719182	2.216406	-0.000047
H	-3.173741	1.905626	-0.000092
O	1.548111	1.491567	0.000103
C	2.107194	-0.844711	0.000101
H	1.885315	-1.476404	0.872226
C	3.565316	-0.41712	-0.000179
H	1.885093	-1.476781	-0.871674
H	3.834493	0.153997	0.896984
H	3.83413	0.153899	-0.897504
H	4.207539	-1.300239	-0.000211

Stacked Complexes Coordinates: abbreviated as Naph/Pyr•••**a**/**b**/**c**/**d**. All complexes involve the protonated form of the ketones. Unless specified otherwise, the coordinates are that of the DFT-optimized structures. The initial coordinates are only provided for Naph/Pyr•••**a** for reference.

*Pre-optimized, Initial Coordinates for Naph•••**a***

C	7.8513	1.1201	9.73113

C	9.11767	1.05737	9.10397
C	10.29132	1.01047	9.86196
C	10.23046	1.02337	11.25401
C	8.99422	1.08633	11.89397
C	7.81554	1.13618	11.14395
H	9.23052	1.04394	8.03174
H	11.25209	0.96179	9.36584
H	11.14172	0.98334	11.83656
C	6.56991	1.16608	8.93856
H	6.8802	1.18153	11.68394
H	8.948	1.09442	12.97538
O	6.54762	1.09771	7.71269
C	5.24081	1.29371	9.63227
H	5.07968	0.42963	10.30984
H	4.4107	1.31293	8.89334
H	5.20933	2.23818	10.21484
C	6.62938	-2.20938	8.09137
C	6.55788	-2.24516	9.48679
C	7.86929	-2.25726	7.4487
C	9.04694	-2.34325	8.19658
C	8.98967	-2.37997	9.59825
C	7.73233	-2.32921	10.24999
H	7.9177	-2.2271	6.36793
H	9.99931	-2.3787	7.68128
C	7.67529	-2.36029	11.65167
C	8.85323	-2.44167	12.39974
C	10.16429	-2.46286	10.36146
C	10.09306	-2.49331	11.75703
H	8.80518	-2.46383	13.48068
H	11.13431	-2.50135	9.88046
H	11.00144	-2.55536	12.34205
H	6.72314	-2.31964	12.16693
H	5.58799	-2.20399	9.96765
H	5.72128	-2.14147	7.50654
H	7.32219	1.00556	7.14663

*Naph•••**a***

C	-1.219071	1.164461	0.194616
C	-1.01747	1.528374	-1.157551

C	0.074687	2.289874	-1.520582
C	0.993371	2.691091	-0.549445
C	0.810703	2.347564	0.790533
C	-0.27637	1.584022	1.161791
H	-1.698385	1.210492	-1.946293
H	0.223795	2.564583	-2.560423
H	1.855306	3.286294	-0.839553
C	-2.346424	0.393441	0.596862
H	-0.410216	1.322488	2.207155
H	1.529297	2.667123	1.538944
O	-3.319207	0.09824	-0.216066
C	-2.61998	-0.045474	1.982265
H	-1.711097	-0.314447	2.522556
H	-3.311022	-0.891348	1.968491
H	-3.110964	0.776528	2.521841
C	-1.215027	-2.31392	-0.590191
C	-0.450583	-2.118167	0.538527
C	-0.761517	-1.854531	-1.844938
C	0.450486	-1.212554	-1.945135
C	1.26248	-1.000838	-0.802585
C	0.800325	-1.459169	0.465959
H	-1.359692	-2.035375	-2.735046
H	0.816213	-0.870896	-2.912926
C	1.599577	-1.231564	1.611279
C	2.811213	-0.589899	1.503938
C	2.510657	-0.342037	-0.880715
C	3.271816	-0.14511	0.248154
H	3.425859	-0.436358	2.387096
H	2.866719	-0.007903	-1.854376
H	4.238302	0.346683	0.173993
H	1.249707	-1.596115	2.576812
H	-0.782349	-2.506284	1.501634
H	-2.163474	-2.842394	-0.519414
H	-3.177573	0.42762	-1.118652

*Naph•••**b***

C	0.872732	-1.407112	0.318875
C	0.430651	-1.797492	-0.970533
C	-0.897169	-2.060631	-1.213113
C	-1.805432	-1.910898	-0.165064
C	-1.414332	-1.555853	1.124785

C	-0.08566	-1.294274	1.35769
H	1.117616	-1.892537	-1.810019
H	-1.258732	-2.35492	-2.193215
F	-3.0783	-2.12583	-0.401753
C	2.236333	-1.117377	0.583666
H	0.222317	-1.004733	2.357669
H	-2.165098	-1.46554	1.903044
O	3.178665	-1.289464	-0.299791
C	2.774051	-0.705476	1.899284
H	2.094826	-0.045097	2.440622
H	3.737893	-0.210923	1.759879
H	2.944163	-1.602166	2.511093
C	2.172134	1.735946	-0.804069
C	1.443069	1.965294	0.340565
C	1.521271	1.356367	-1.998168
C	0.154003	1.215515	-2.021922
C	-0.62423	1.444189	-0.859134
C	0.033904	1.825692	0.346902
H	2.102328	1.20578	-2.905216
H	-0.357616	0.940966	-2.943965
C	-0.739143	2.039521	1.513076
C	-2.105638	1.887363	1.48644
C	-2.029393	1.28722	-0.853324
C	-2.7559	1.506913	0.2946
H	-2.691537	2.070895	2.383329
H	-2.530241	1.003531	-1.778466
H	-3.837242	1.396723	0.282891
H	-0.234225	2.348819	2.427836
H	1.940268	2.293298	1.253518
H	3.252114	1.86761	-0.79681
H	2.851035	-1.592973	-1.161423

*Naph•••**c***

C	1.176783	-0.974778	0.398021
C	0.768649	-1.59709	-0.815083
C	-0.457077	-2.169271	-1.014196
C	-1.371918	-2.096778	0.036681
C	-1.067967	-1.512847	1.260582
C	0.176209	-0.952086	1.410988

F	1.649565	-1.660189	-1.822001
H	-0.712691	-2.638891	-1.957885
F	-2.559612	-2.618193	-0.134502
C	2.464936	-0.39365	0.579976
F	0.45208	-0.37095	2.561291
H	-1.800206	-1.459406	2.058531
O	3.361493	-0.402893	-0.355973
C	2.968058	0.195983	1.839958
H	2.337177	1.030562	2.156063
H	3.992502	0.537459	1.686429
H	2.941277	-0.544436	2.646973
C	1.509109	2.316403	-0.68066
C	0.587056	2.388658	0.338483
C	1.161211	1.721823	-1.914999
C	-0.098503	1.20704	-2.101056
C	-1.069226	1.264813	-1.068606
C	-0.717639	1.864054	0.177504
H	1.888221	1.694492	-2.723707
H	-0.378408	0.760838	-3.055027
C	-1.680762	1.908021	1.216238
C	-2.936334	1.383415	1.029731
C	-2.367279	0.726261	-1.227209
C	-3.28204	0.78582	-0.201815
H	-3.673058	1.43566	1.827067
H	-2.636414	0.27786	-2.182985
H	-4.281108	0.380627	-0.340613
H	-1.410572	2.376816	2.161321
H	0.839832	2.875955	1.280548
H	2.501921	2.742579	-0.549298
H	3.041689	-0.797725	-1.18994

Naph•••d

C	-1.13711	1.23785	0.14704
C	-0.89018	1.57046	-1.19744
C	0.20129	2.36951	-1.5472
C	1.06732	2.84117	-0.56028
C	0.83864	2.52014	0.77875
C	-0.25745	1.72834	1.13617
H	-1.54284	1.20603	-1.98116

H	0.37878	2.61672	-2.58579
H	1.91751	3.45239	-0.8336
C	-2.31998	0.33752	0.45488
H	-0.39251	1.49827	2.18262
H	1.51571	2.88124	1.54196
O	-3.48273	0.84035	-0.13559
C	-2.57321	0.0101	1.93608
H	-1.64013	-0.3556	2.4131
C	-3.61142	-1.09832	2.08927
H	-2.91877	0.92526	2.46468
C	-1.1291	-2.44615	-0.63144
C	-0.35836	-2.2402	0.51702
C	-0.69128	-1.96338	-1.86757
C	0.52163	-1.2767	-1.96522
C	1.30334	-1.05916	-0.81948
C	0.85544	-1.54376	0.43537
H	-1.29587	-2.11999	-2.75148
H	0.84431	-0.90766	-2.93157
C	1.62965	-1.31437	1.58339
C	2.83584	-0.61583	1.48796
C	2.51696	-0.36179	-0.90122
C	3.27939	-0.1424	0.24982
H	3.42931	-0.44	2.3759
H	2.87415	0.01655	-1.85178
H	4.21452	0.39763	0.18263
H	1.30013	-1.67205	2.55186
H	-0.71667	-2.61524	1.4684
H	-2.0711	-2.97375	-0.56181
H	-3.66159	0.22034	-0.88914
H	-4.58065	-0.80421	1.6357
H	-3.77293	-1.30871	3.16777
H	-3.24991	-2.02869	1.60199

*Pre-optimized, Initial Coordinates for Pyr•••**a***

C	6.97172	1.88003	9.44187
C	8.24096	1.79274	8.83359
C	9.39146	1.62656	9.61054
C	9.29459	1.54776	11.00042
C	8.04619	1.63366	11.61763

C	6.89166	1.7967	10.8467
H	8.35452	1.84795	7.75977
H	10.36025	1.55722	9.13312
H	10.18674	1.41787	11.5991
C	5.73042	2.05369	8.63206
H	5.93636	1.85728	11.35267
H	7.97163	1.57022	12.69551
O	4.64506	2.08529	9.20422
C	5.79285	2.19213	7.12868
H	6.39285	3.08706	6.86207
H	4.78106	2.30938	6.68619
H	6.2612	1.29117	6.68295
C	4.26376	-1.24871	7.85912
C	4.18353	-1.25436	9.255
C	5.34343	-1.41047	10.02804
C	5.49813	-1.39772	7.21973
C	6.67022	-1.55215	7.97448
C	6.59275	-1.56034	9.38517
H	3.21647	-1.13611	9.72876
H	3.36448	-1.12691	7.26943
H	5.53651	-1.38837	6.13703
C	7.91677	-1.69645	7.34715
C	9.07999	-1.84899	8.10905
C	9.01514	-1.86029	9.51046
C	7.76526	-1.71538	10.1533
H	7.99238	-1.69057	6.26638
H	10.03114	-1.95787	7.60179
C	7.68788	-1.72246	11.56416
C	6.44182	-1.57306	12.19148
C	5.27903	-1.41761	11.42948
H	6.36677	-1.57428	13.27227

*Pyr•••**a***

C	-0.260585	1.889893	-0.898621
C	-1.031579	2.778116	-0.115904
C	-2.40889	2.68762	-0.125288
C	-3.034092	1.712294	-0.905698
C	-2.286745	0.831196	-1.68866
C	-0.908956	0.907844	-1.683214

H	-0.549061	3.538929	0.491748
H	-3.003565	3.370826	0.473204
H	-4.118914	1.64081	-0.905713
C	1.159632	1.952183	-0.870485
H	-0.315702	0.213218	-2.271766
H	-2.785752	0.073772	-2.285556
O	1.793148	1.149671	-1.670131
C	1.923029	2.940296	-0.07069
H	1.769239	3.93783	-0.503966
H	2.993939	2.724476	-0.075806
H	1.572054	2.97025	0.964802
C	3.715177	-0.511758	0.3159
C	3.124614	-1.434811	-0.547447
C	1.757762	-1.728439	-0.458041
C	2.95197	0.135821	1.282112
C	1.575168	-0.112584	1.394701
C	0.970037	-1.054997	0.516007
H	3.729366	-1.95425	-1.288805
H	4.785304	-0.325837	0.256784
H	3.421291	0.834038	1.974732
C	0.755533	0.554888	2.364371
C	-0.582118	0.328487	2.425413
C	-1.221934	-0.600895	1.540945
C	-0.426976	-1.29906	0.591111
H	1.234539	1.24209	3.061325
H	-1.196049	0.839888	3.165109
C	-1.034163	-2.229434	-0.295924
C	-0.211574	-2.909962	-1.251138
C	1.122248	-2.668502	-1.332369
H	-0.682828	-3.631265	-1.916098
H	1.736263	-3.190858	-2.063264
C	-2.415932	-2.441438	-0.215206
C	-2.599242	-0.83402	1.577167
C	-3.18736	-1.749036	0.708868
H	-2.879258	-3.163604	-0.885008
H	-3.207309	-0.301398	2.3065
H	-4.257607	-1.931152	0.761797
H	2.738715	1.052655	-1.446431

*Pyr•••**b***

C	-0.303919	1.768277	-0.86675
C	-1.133583	2.496361	0.01992
C	-2.474562	2.20427	0.118824
C	-2.989842	1.176105	-0.670571
C	-2.21308	0.45354	-1.576399
C	-0.870585	0.736073	-1.657293
H	-0.721339	3.293121	0.632494
H	-3.135294	2.738705	0.79368
F	-4.265264	0.879897	-0.561289
C	1.086831	2.026679	-0.927023
H	-0.233201	0.160326	-2.322912
H	-2.67464	-0.334913	-2.161898
O	1.77453	1.321286	-1.777289
C	1.761567	3.108709	-0.168684
H	1.477208	4.076378	-0.603737
H	2.849414	3.016854	-0.217454
H	1.459084	3.113077	0.881724
C	3.954943	-0.074331	0.257633
C	3.460411	-1.058448	-0.597455
C	2.140507	-1.51351	-0.480702
C	3.14058	0.47621	1.241367
C	1.807069	0.062743	1.384126
C	1.299987	-0.943625	0.51438
H	4.106713	-1.501701	-1.353225
H	4.993101	0.239654	0.175815
H	3.537297	1.225686	1.925716
C	0.935867	0.622891	2.375632
C	-0.362996	0.235442	2.466766
C	-0.907219	-0.760798	1.591113
C	-0.055143	-1.355953	0.620694
H	1.34392	1.361678	3.06488
H	-1.016622	0.666744	3.223581
C	-0.566861	-2.347303	-0.260512
C	0.30949	-2.92013	-1.238308
C	1.602561	-2.519952	-1.346726
H	-0.087101	-3.689512	-1.898087
H	2.257907	-2.962221	-2.094445
C	-1.914109	-2.716808	-0.157542

C	-2.248317	-1.149937	1.649152
C	-2.744288	-2.120438	0.782236
H	-2.304191	-3.482871	-0.825263
H	-2.901187	-0.69397	2.392042
H	-3.787574	-2.417979	0.847595
H	2.735798	1.385186	-1.633854

*Pyr***•••*c***

C	0.096327	1.603608	0.867932
C	0.969051	2.348137	0.025609
C	2.320761	2.110284	-0.06318
C	2.851625	1.075676	0.697068
C	2.077535	0.299062	1.557998
C	0.73672	0.563019	1.597195
F	0.474222	3.334525	-0.696138
H	2.94508	2.706586	-0.7196
F	4.135253	0.828411	0.61688
C	-1.299537	1.886669	0.975477
F	-0.018494	-0.19966	2.395228
H	2.507709	-0.51295	2.133698
O	-2.090891	1.189681	1.727357
C	-1.97669	3.047299	0.355115
H	-1.551593	3.981086	0.742144
H	-3.041035	3.001684	0.589555
H	-1.829452	3.061755	-0.727267
H	-1.661377	0.408881	2.127005
C	-3.897907	-0.277359	-0.527078
C	-3.438878	-1.312818	0.281537
C	-2.105743	-1.739523	0.216709
C	-3.035934	0.361207	-1.407459
C	-1.688122	-0.020129	-1.498315
C	-1.215979	-1.084798	-0.679451
H	-4.121999	-1.818048	0.962052
H	-4.940114	0.025584	-0.47407
H	-3.403358	1.158032	-2.053258
C	-0.77206	0.617424	-2.397494
C	0.531514	0.238415	-2.456553
C	1.037893	-0.823765	-1.636606
C	0.144692	-1.48688	-0.749913

H	-1.14729	1.410691	-3.043074
H	1.217751	0.727116	-3.147058
C	0.616904	-2.545318	0.075066
C	-0.3067	-3.202506	0.950374
C	-1.607181	-2.816975	1.020255
H	0.058509	-4.025718	1.561354
H	-2.300479	-3.328435	1.685372
C	1.970651	-2.902781	0.010834
C	2.381878	-1.20655	-1.659183
C	2.841026	-2.237182	-0.841691
H	2.331379	-3.718745	0.634407
H	3.066548	-0.699667	-2.337941
H	3.886946	-2.530076	-0.881386

*Pyr•••**d***

C	0.349631	1.739508	-0.861937
C	-0.105707	2.755882	0.007893
C	-1.433258	3.133705	-0.008071
C	-2.321929	2.510277	-0.885887
C	-1.885702	1.510564	-1.757555
C	-0.565049	1.112513	-1.739275
H	0.579541	3.243947	0.695451
H	-1.785197	3.911931	0.662384
H	-3.366561	2.811351	-0.895587
C	1.713061	1.335227	-0.84426
H	-0.221451	0.315651	-2.393515
H	-2.587856	1.031086	-2.43286
O	2.049191	0.380965	-1.657033
C	2.760023	1.998851	-0.012694
H	2.686334	3.077363	-0.211023
C	4.179918	1.514519	-0.246719
H	2.466793	1.876699	1.040415
C	2.981377	-2.005464	0.511841
C	2.129206	-2.587676	-0.423082
C	0.759091	-2.298175	-0.421687
C	2.487919	-1.106193	1.450676
C	1.124758	-0.773503	1.478933
C	0.247166	-1.384018	0.5397
H	2.517684	-3.299157	-1.149728

H	4.036132	-2.272846	0.521928
H	3.154445	-0.66708	2.19343
C	0.583923	0.153606	2.429027
C	-0.738934	0.464806	2.429886
C	-1.646513	-0.136015	1.49753
C	-1.137506	-1.068455	0.552817
H	1.256858	0.592242	3.165553
H	-1.140918	1.167041	3.158784
C	-2.013397	-1.674649	-0.388155
C	-1.471006	-2.597307	-1.340494
C	-0.145862	-2.892824	-1.35987
H	-2.150737	-3.06387	-2.051083
H	0.25314	-3.599208	-2.0852
C	-3.374103	-1.343651	-0.360798
C	-3.008772	0.175764	1.479413
C	-3.863458	-0.428535	0.561412
H	-4.047292	-1.81927	-1.071808
H	-3.398632	0.887008	2.205991
H	-4.9236	-0.188761	0.573041
H	4.509053	1.681534	-1.279028
H	4.298984	0.453871	0.010247
H	4.869714	2.066935	0.394913
H	2.970784	0.093281	-1.52791

Chiral HPLC Traces

Traces of the racemic and enantioenriched products are provided. For the host-catalyzed products, traces with hosts **1** and **2** are provided when applicable for the reactions run at 35 °C.

Datafile Name:phenylethanol-rac_IB_9505_30min_1mL_022.lcd
Sample Name:phenylethanol-rac

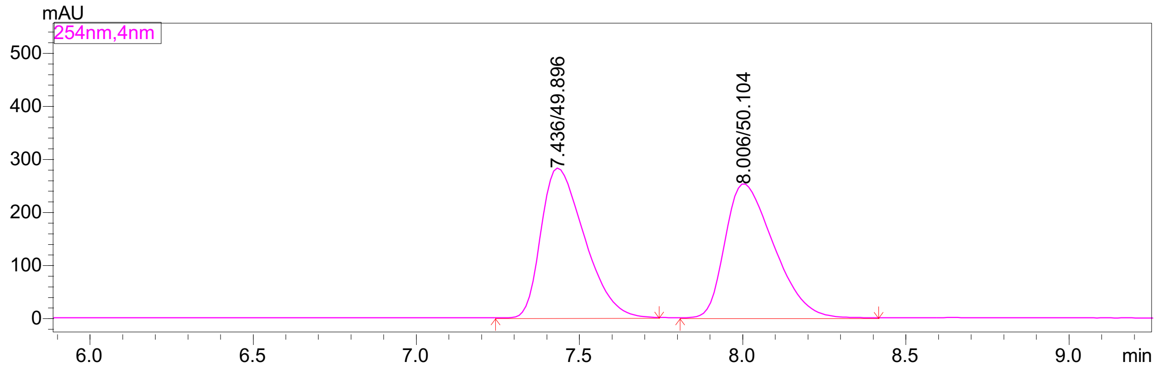

Datafile Name:CD-2-30-65N_IB_9505_30min_1mL_016.lcd
Sample Name:CD-2-30-65N

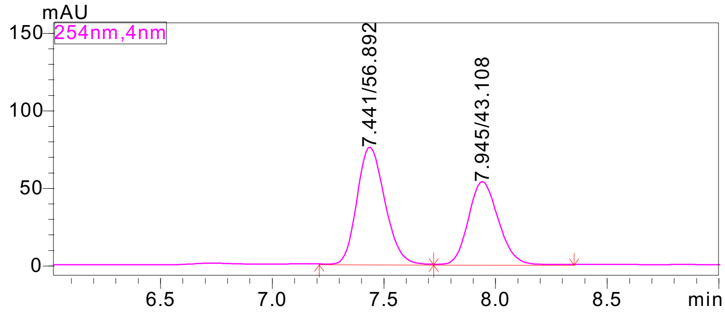

Datafile Name:CD-2-29-35P_IB_9505_30min_1mL_007.lcd
Sample Name:CD-2-29-35P

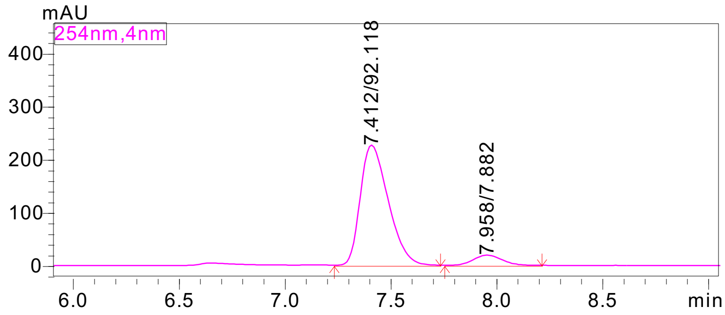

Figure S25. Racemic (top) and enantioenriched traces (**1** middle, **2** bottom) alcohol **a1**

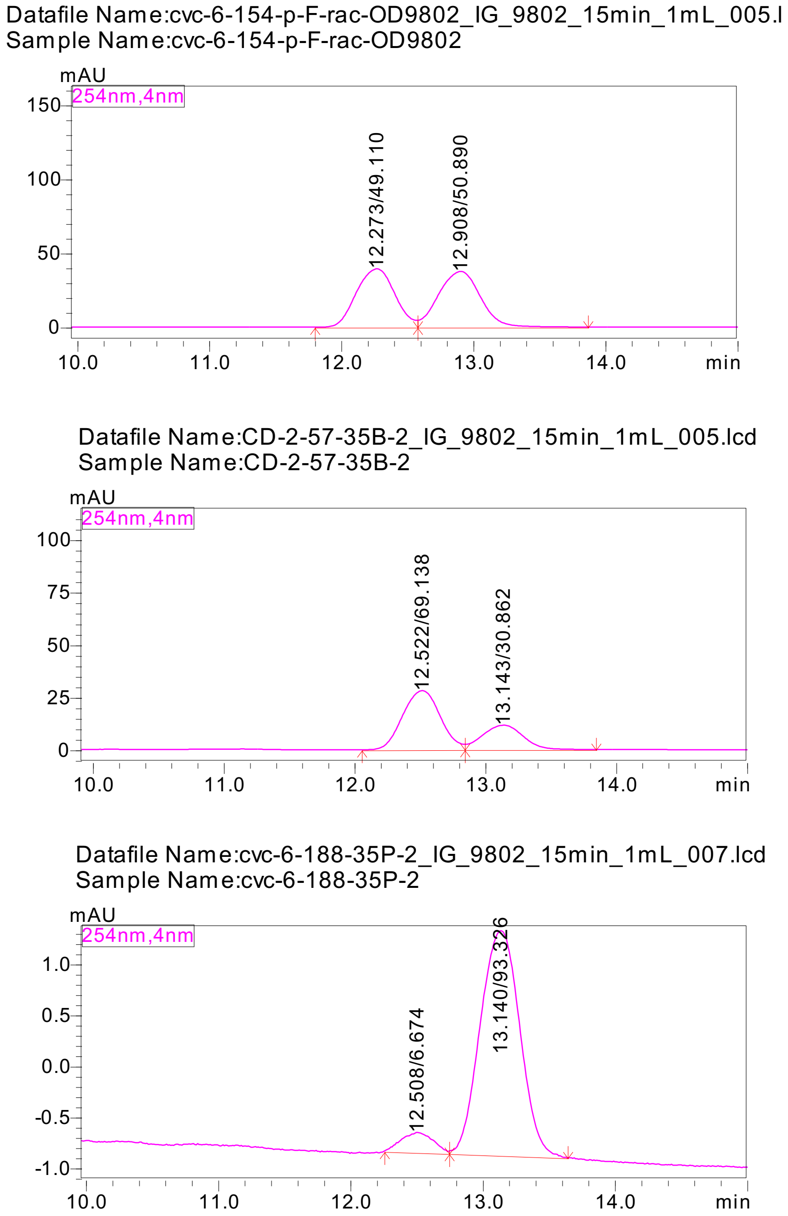

Figure S26. Racemic (top) and enantioenriched traces (**1** middle, **2** bottom) alcohol **b1**

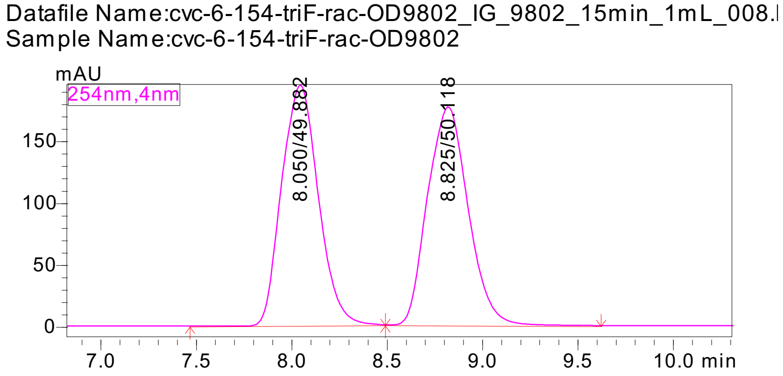

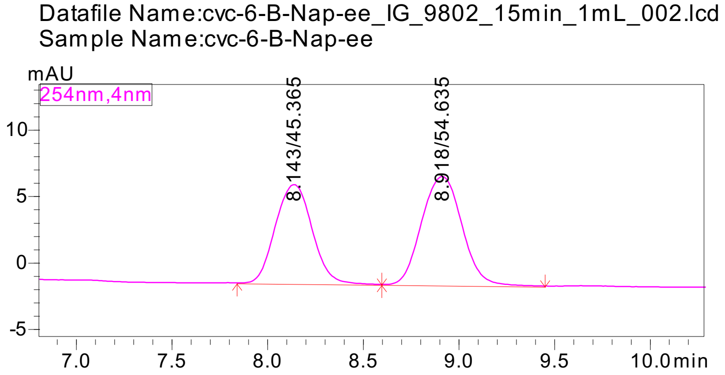

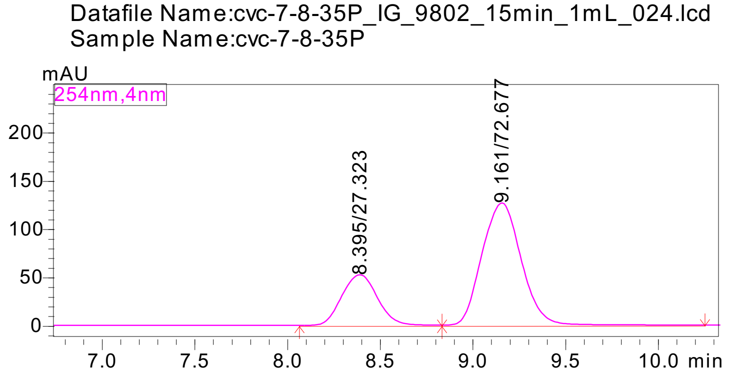

Figure S27. Racemic (top) and enantioenriched traces (**1** middle, **2** bottom) alcohol **c1**

Datafile Name:cvc-6-172-rac-PrOH-4_IG_9901_30min_1mL_013.lcc
Sample Name:cvc-6-172-rac-PrOH-4

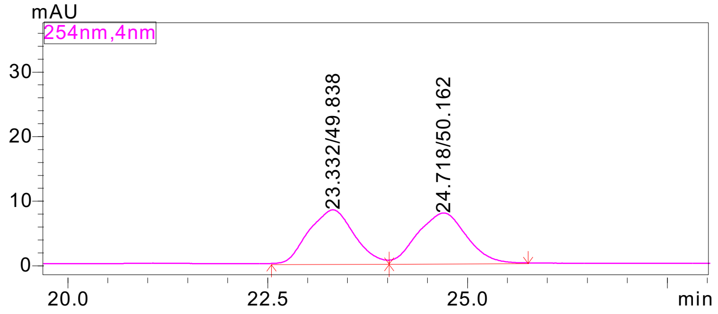

Datafile Name:CD-2-61-35A_IG_9901_30min_1mL_004.lcd
Sample Name:CD-2-61-35A

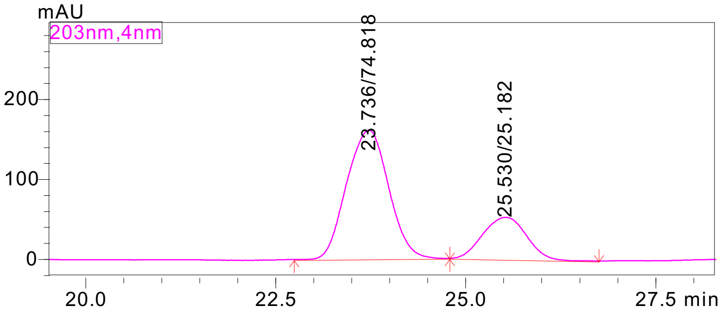

Datafile Name:cvc-6-201-35A_IG_9901_30min_1mL_006.lcd
Sample Name:cvc-6-201-35A

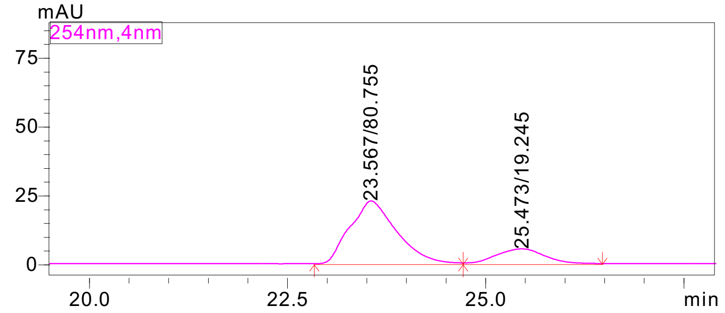

Figure S28. Racemic (top) and enantioenriched traces (**1** middle, **2** bottom) alcohol **d1**

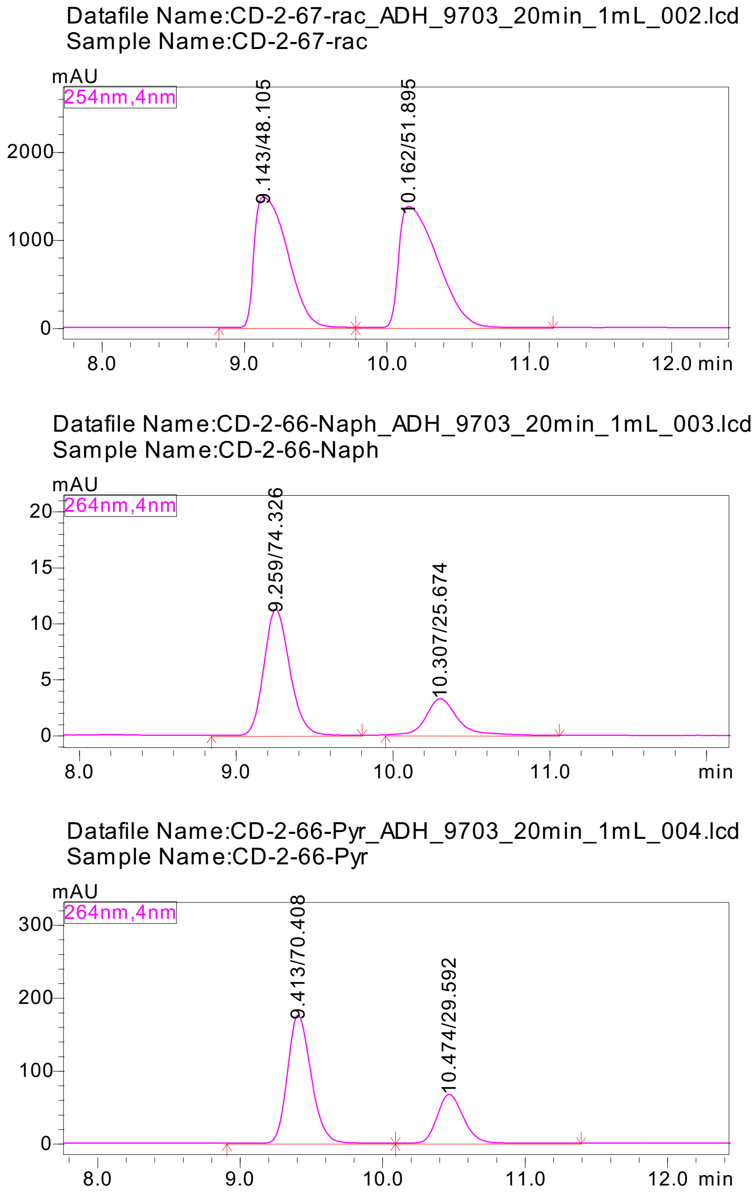

Figure S29. Racemic (top) and enantioenriched traces (**1** middle, **2** bottom) alcohol **e1**

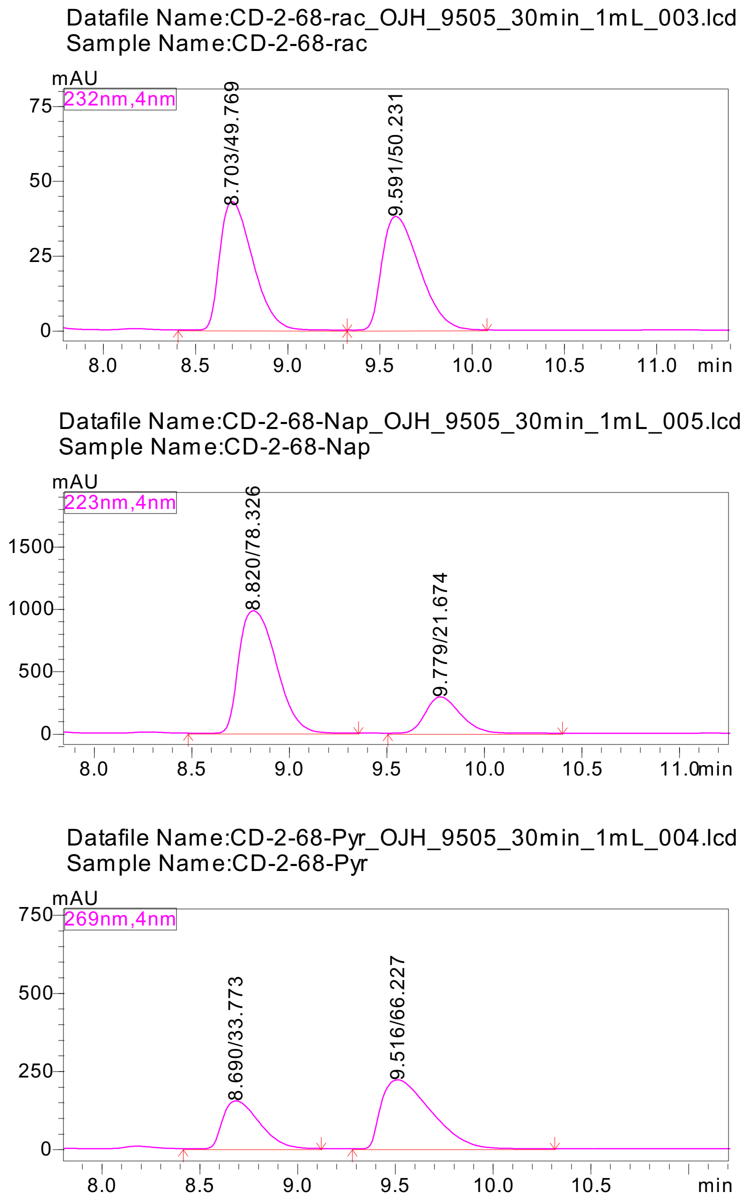

Figure S30. Racemic (top) and enantioenriched traces (**1** middle, **2** bottom) alcohol **f1**

Datafile Name:cvc-6-180-rac-3_IC_9802_30min_0.8mL_003.lcd
Sample Name:cvc-6-180-rac-3

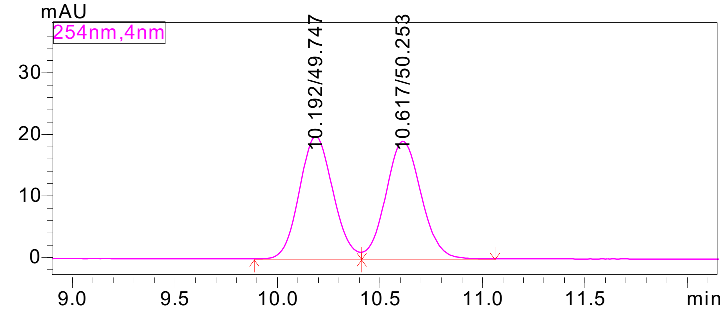

Datafile Name:CD-2-50-A-Nap-2_IC_9802_30min_0.8mL_004.lcd
Sample Name:CD-2-50-A-Nap-2

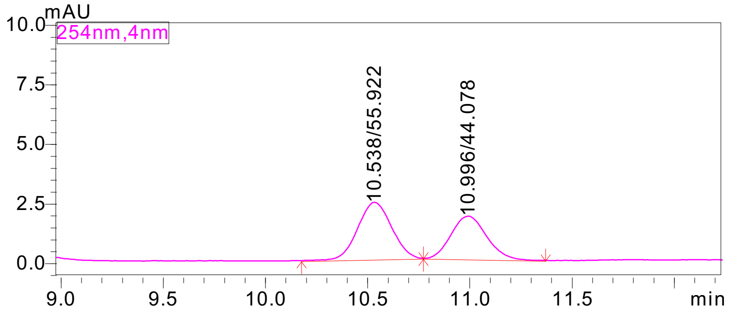

Datafile Name:CD-2-50-A-Pyr-2_IC_9802_30min_0.8mL_005.lcd
Sample Name:CD-2-50-A-Pyr-2

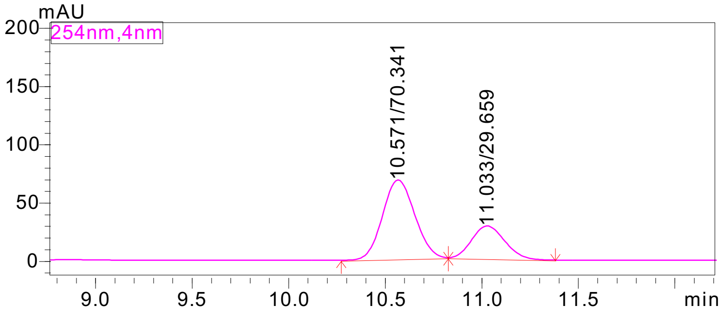

Figure S31. Racemic (top) and enantioenriched traces (**1** middle, **2** bottom) alcohol **g1**

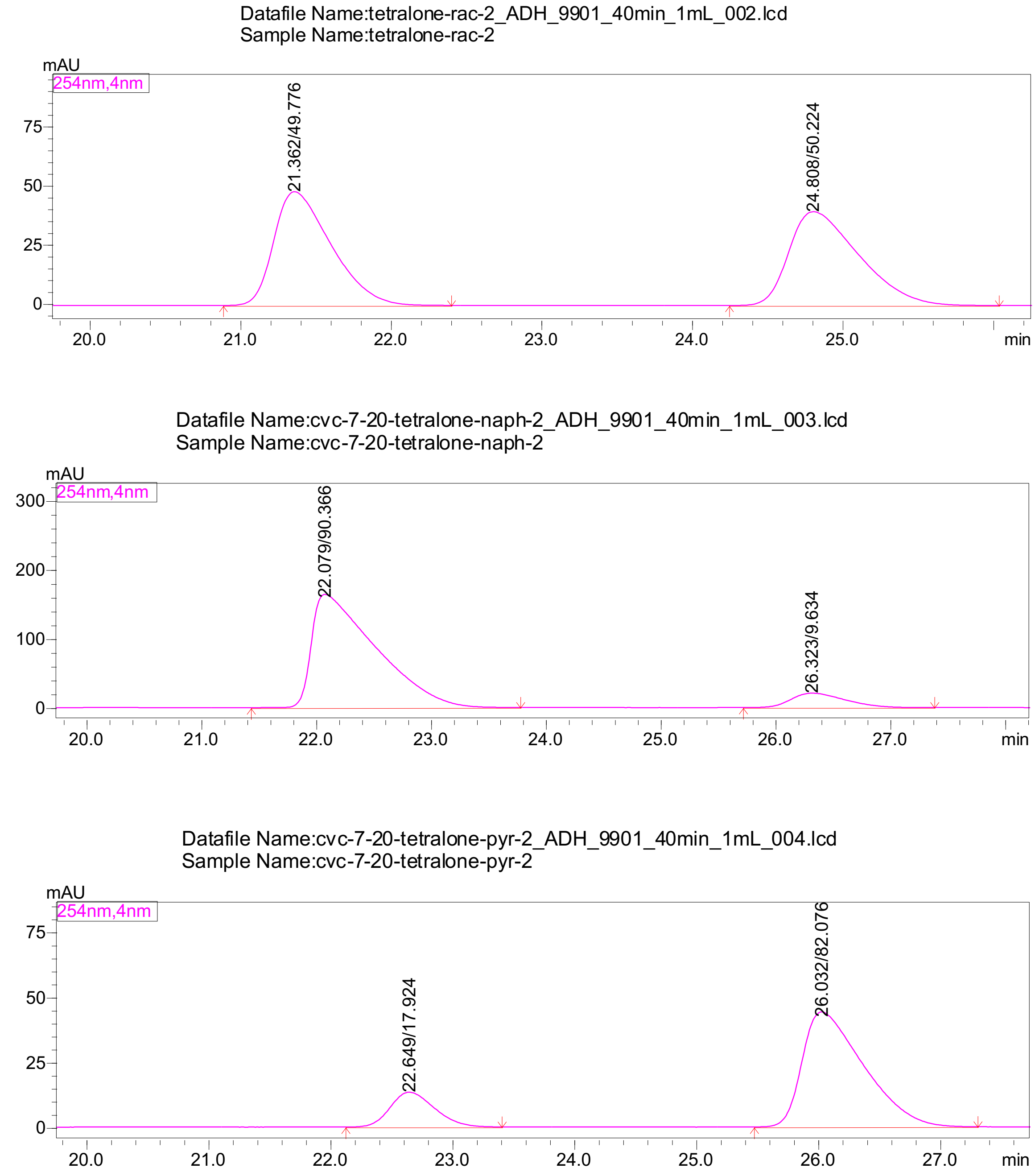

Figure S32. Racemic (top) and enantioenriched traces (**1** middle, **2** bottom) alcohol **h1**

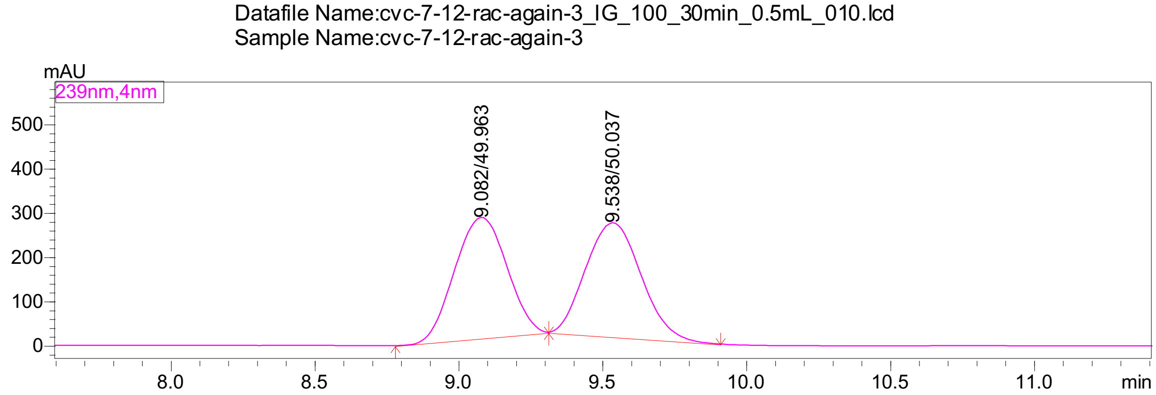

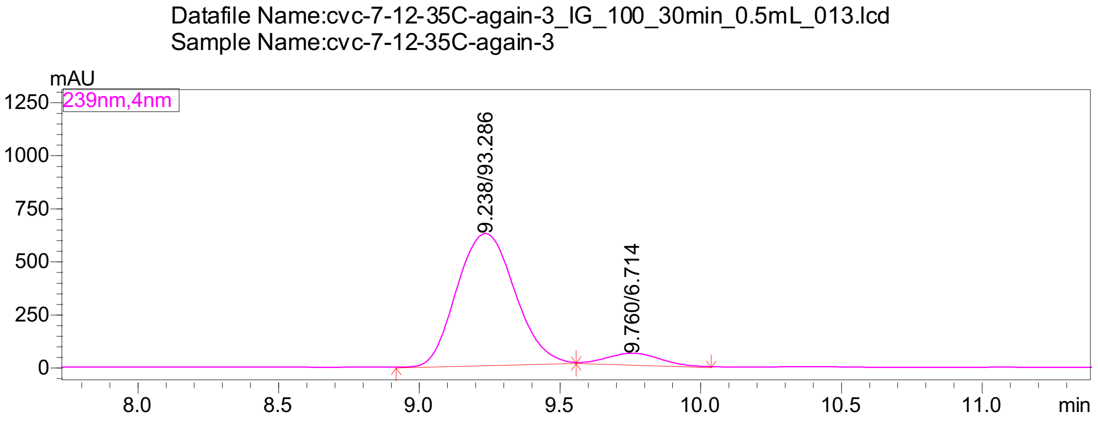

Figure S33. Racemic (top) and enantioenriched trace (**2** bottom) benzoyl-functionalized alcohol **i1**

Supplemental References

(1) Bierschenk, S. M.; Pan, J. Y.; Settineri, N. S.; Warzok, U.; Bergman, R. G.; Raymond, K. N.; Toste, F. D. Impact of Host Flexibility on Selectivity in a Supramolecular Host-Catalyzed Enantioselective Aza-Darzens Reaction. *J. Am. Chem. Soc.* **2022**, *144* (25), 11425–11433. https://doi.org/10.1021/jacs.2c04182.

(2) Ling, F.; Nian, S.; Chen, J.; Luo, W.; Wang, Z.; Lv, Y.; Zhong, W. Development of Ferrocene-Based Diamine-Phosphine-Sulfonamide Ligands for Iridium-Catalyzed Asymmetric Hydrogenation of Ketones. *J. Org. Chem.* **2018**, *83* (18), 10749–10761. https://doi.org/10.1021/acs.joc.8b01276.

(3) Cambeiro, X. C.; Ahlsten, N.; Larrosa, I. Au-Catalyzed Cross-Coupling of Arenes via Double C–H Activation. *J. Am. Chem. Soc.* **2015**, *137* (50), 15636–15639. https://doi.org/10.1021/jacs.5b10593.

(4) Williams, M. T. J.; Adarve Cardona, L.; Bolm, C. Mechanochemical Mitsunobu Reactions. *Adv. Synth. Catal.* **2024**, *366* (10), 2220–2225. https://doi.org/10.1002/adsc.202400296.

(5) Davis, M. R.; Dougherty, D. A. Cation–π Interactions: Computational Analyses of the Aromatic Box Motif and the Fluorination Strategy for Experimental Evaluation. *Phys. Chem. Chem. Phys.* **2015**, *17* (43), 29262–29270. https://doi.org/10.1039/C5CP04668H.
